# Supplementary material for: Automatic optimization of flat-field corrections by evaluation and enhancement (EVEN) in multimodal optical microscopy
Source: Nat Commun. 2026 Jan 7;17:225. doi: 10.1038/s41467-025-68150-0 (PMC12779969; doi:10.1038/s41467-025-68150-0)
Supplement: Supplementary file 1 — Supplementary information [file 41467_2025_68150_MOESM1_ESM.pdf]

Automatic optimization of flat-field corrections by evaluation and enhancement (EVEN) in multimodal optical microscopy – Supplementary Information

Elena Corbetta[1,2,3], Matteo Calvarese[1], Patrick Then[1,6], Hyeonsoo Bae[1], Tobias Meyer-Zedler[1], Bernhard Messerschmidt[4], Orlando Guntinas-Lichius[5], Michael Schmitt[2,3], Christian Eggeling[1,6], Juergen Popp[1,2,3], Thomas Bocklitz[1,2,3]*

1. *Leibniz Institute of Photonic Technology, Member of Leibniz Health Technologies, Member of the Leibniz Centre for Photonics in Infection Research (LPI), Albert-Einstein-Strasse 9, 07745 Jena, Germany.*
2. *Institute of Physical Chemistry (IPC), Friedrich Schiller University Jena, Member of the Leibniz Centre for Photonics in Infection Research (LPI), Helmholtzweg 4, 07743 Jena, Germany*
3. *Abbe Center of Photonics (ACP), Friedrich Schiller University Jena, Member of the Leibniz Centre for Photonics in Infection Research (LPI), Helmholtzweg 4, 07743 Jena, Germany*
4. *GRINTECH GmbH, Otto-Eppenstein-Str. 7 Jena, Germany*
5. *Department of Otorhinolaryngology, Jena University Hospital, Am Klinikum 1, 07747 Jena, Germany*
6. *Institute of Applied Optics and Biophysics, Friedrich Schiller University, Philosophenweg 7, 07743 Jena, Germany*

**email: thomas.bocklitz@uni-jena.de*

Supplementary Fig. 1: Subset of the known training dataset

(a) Samples from Bad class

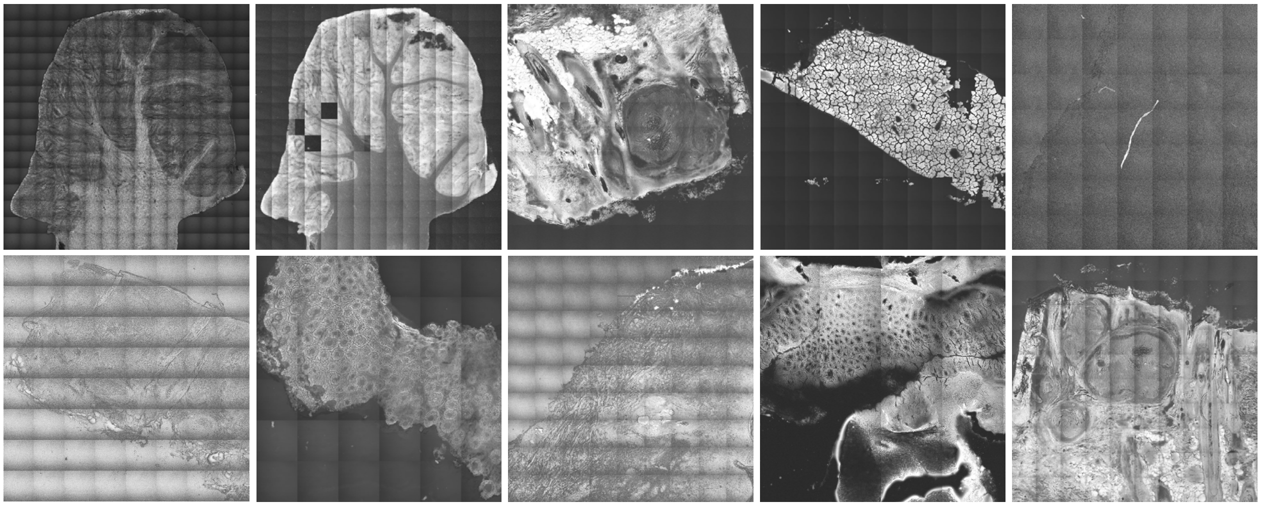

(b) Samples from Good class

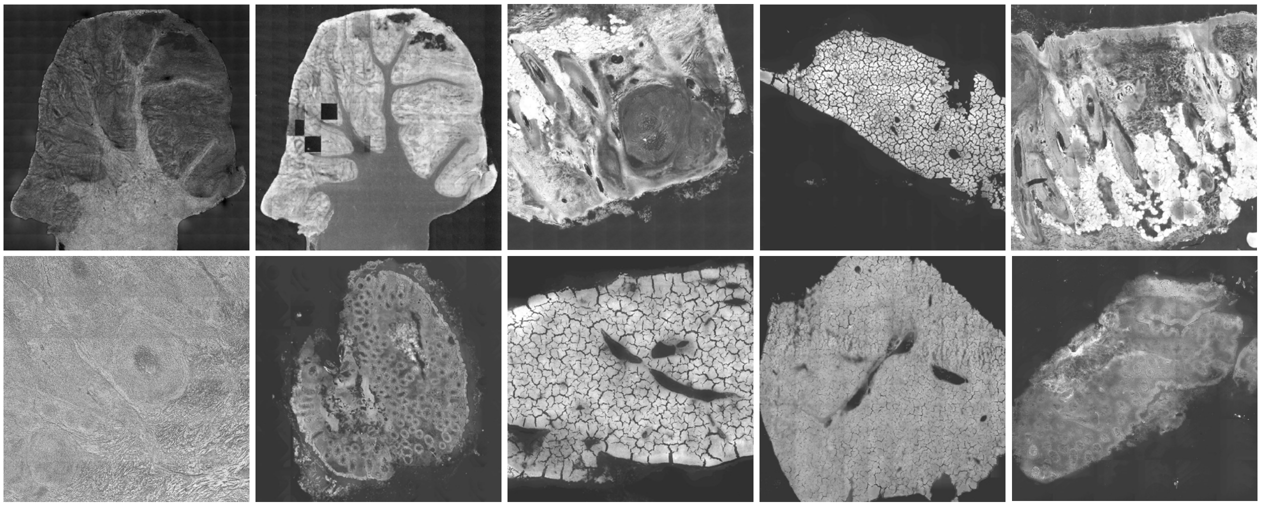

*Supplementary Fig. 1 – **Example images from the training dataset.** Subset of square crops from the training dataset introduced by the previous work of Chernavskaia et al.[1], with tile size equal to 512 px, variable number of tiles and a wide variety of imaged structures. The first four images from the top right are paired in the two classes. (a) Example images from the bad class: experimental uneven illumination. (b) Example images from the good class: good corrections of the experimental measurements.*

Supplementary Fig. 2: LDA coefficients and means

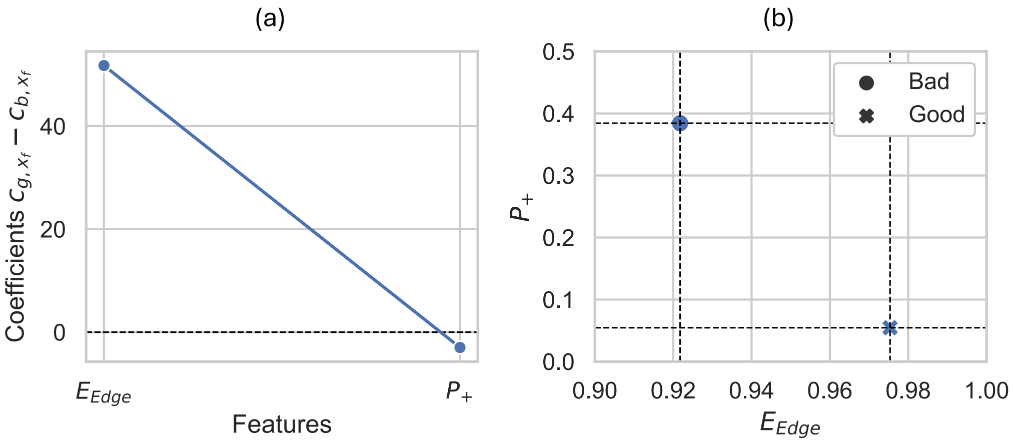

Supplementary Fig. 2 – Weighting coefficients and means of the trained Linear Discriminant Analysis (LDA) model. *(a) Weighting coefficients associated with the quality metrics used as features for model training. A positive value of the coefficient is assigned to features that are higher for images without uneven illumination, and vice versa. (b) Means of the good and bad class. This plot highlights that images without strong artifacts are associated to low positive prominence and high edge energy ratio.*

Supplementary Fig. 3: Comparison between machine learning-based classifiers

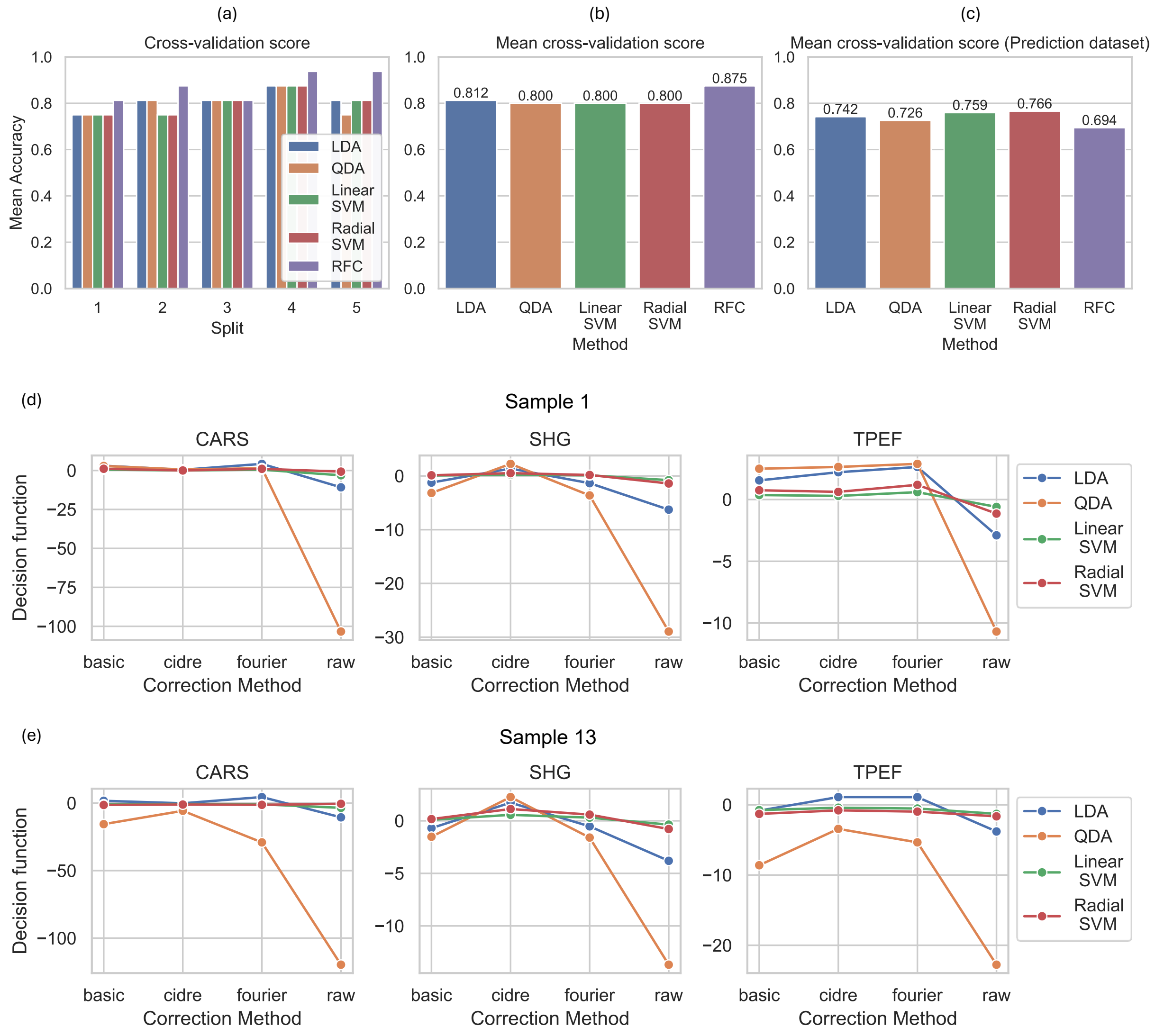

Supplementary Fig. 3 – Comparison between machine learning (ML)-based classifiers. *5-fold cross validation is executed with Linear Discriminant Analysis (LDA), Quadratic Discriminant Analysis (QDA), Linear Support Vector Machine (Linear SVM), radial SVM and Random Forest Classifier (RFC). (a) Mean accuracy for the same splits for each method. Random Forest (RFC) shows the best performance, followed by LDA. (b) Cross-validation accuracy averaged on n = 5 splits. (c) Accuracy of the final models on multimodal nonlinear microscopy measurements of head and neck tissue (Prediction dataset 1). LDA keeps a good performance on the prediction dataset, while RFC shows overfitting. The consistency between quality rankings obtained by LDA and other classifiers is assessed through Kendall's rank correlation coefficient computed between the decision scores predicted by the models. The average correlation coefficients for n = 69 rankings are 0.72 for LDA vs QDA, 0.76 for LDA vs Linear SVM, and 0.68 for LDA vs Radial SVM. We report the decision scores of different classifiers for two samples of Prediction dataset 1: (c) sample 1, (d) sample 13 (Fig. 4 of main paper). The scores show good agreement between different classifiers. Mismatches are found mostly between images with similar quality and linear models show generally better performance and generalizability.*

Supplementary Fig. 4: Decision scores of prediction dataset 1

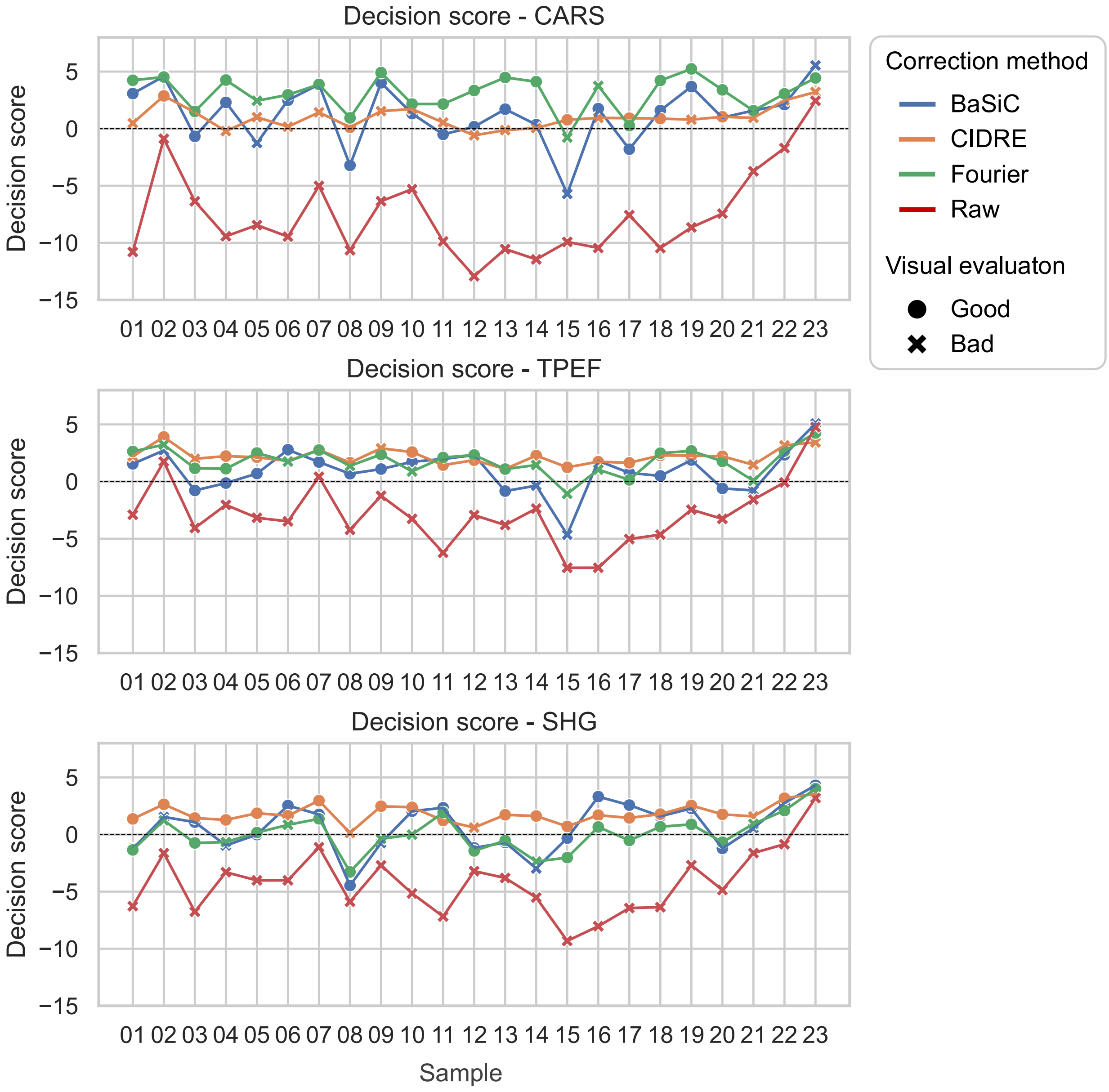

Supplementary Fig. 4 – Decision score of single-channel images for the prediction dataset 1. *The prediction dataset is composed of 23 multimodal measurements (x-axis)[2]. The decision score is computed for single channels of raw and corrected images for each sample: CARS (red channel, top panel), TPEF (green channel, middle panel), SHG (blue channel, bottom panel). Colours indicate the correction method, markers identify our visual evaluation of the image: 'Good' is assigned to high-quality images with good removal of uneven illumination, 'Bad' is assigned to images with strong residual uneven illumination or unwanted processing artifacts.*

Supplementary Fig. 5: Quality metrics of prediction dataset 1

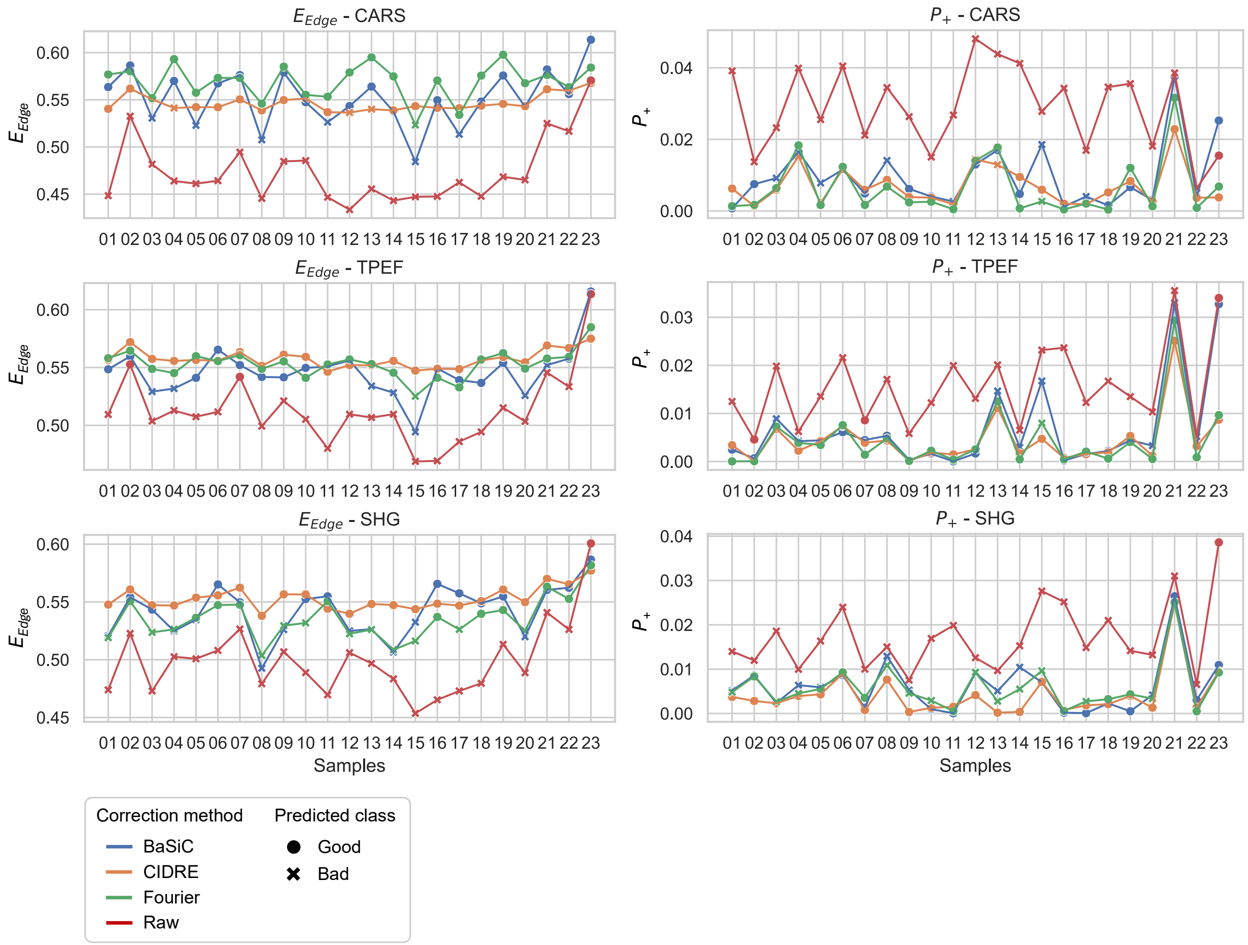

Supplementary Fig. 5 – Quality metrics of prediction dataset 1. *The prediction dataset is composed of 23 multimodal measurements (x-axis)[2]. This figure displays the edge energy ratio (E_{edge}, left column) and the positive prominence (P_+, right column) computed for the three channels of each measurement and each correction: CARS (red channel, top panel), TPEF (green channel, middle panel), SHG (blue channel, bottom panel). Colours indicate the correction method and markers identify the class predicted by the trained model.*

Supplementary Fig. 6 - Fig. 28: Optimization of uneven illumination correction for prediction dataset 1

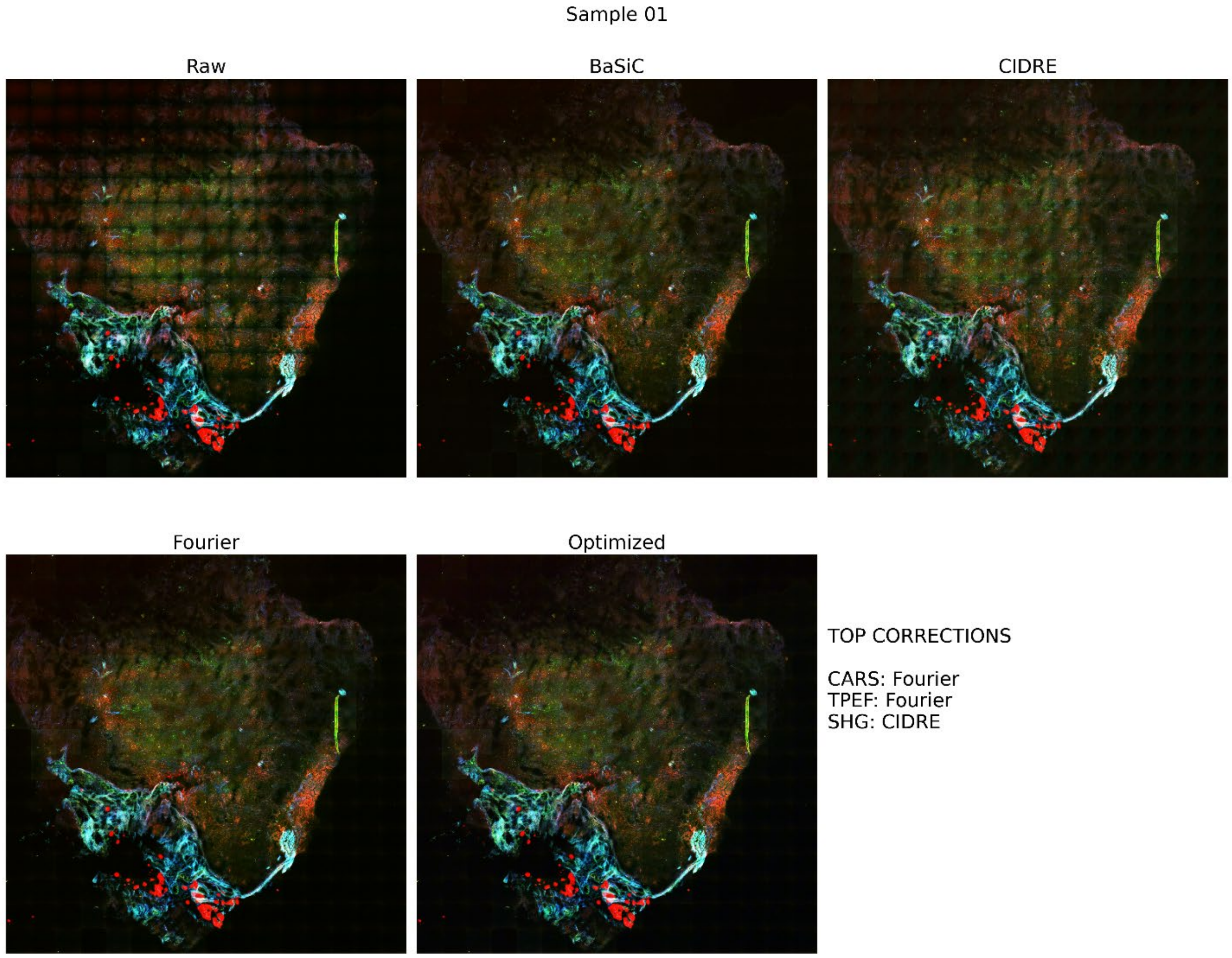

Supplementary Fig. 6 – Single-method corrections and EVEN output (optimized) for Sample 01. *Red channel: coherent anti-Stokes Raman scattering (CARS); green channel: two-photon excited fluorescence (TPEF); blue channel: second harmonic generation (SHG).*

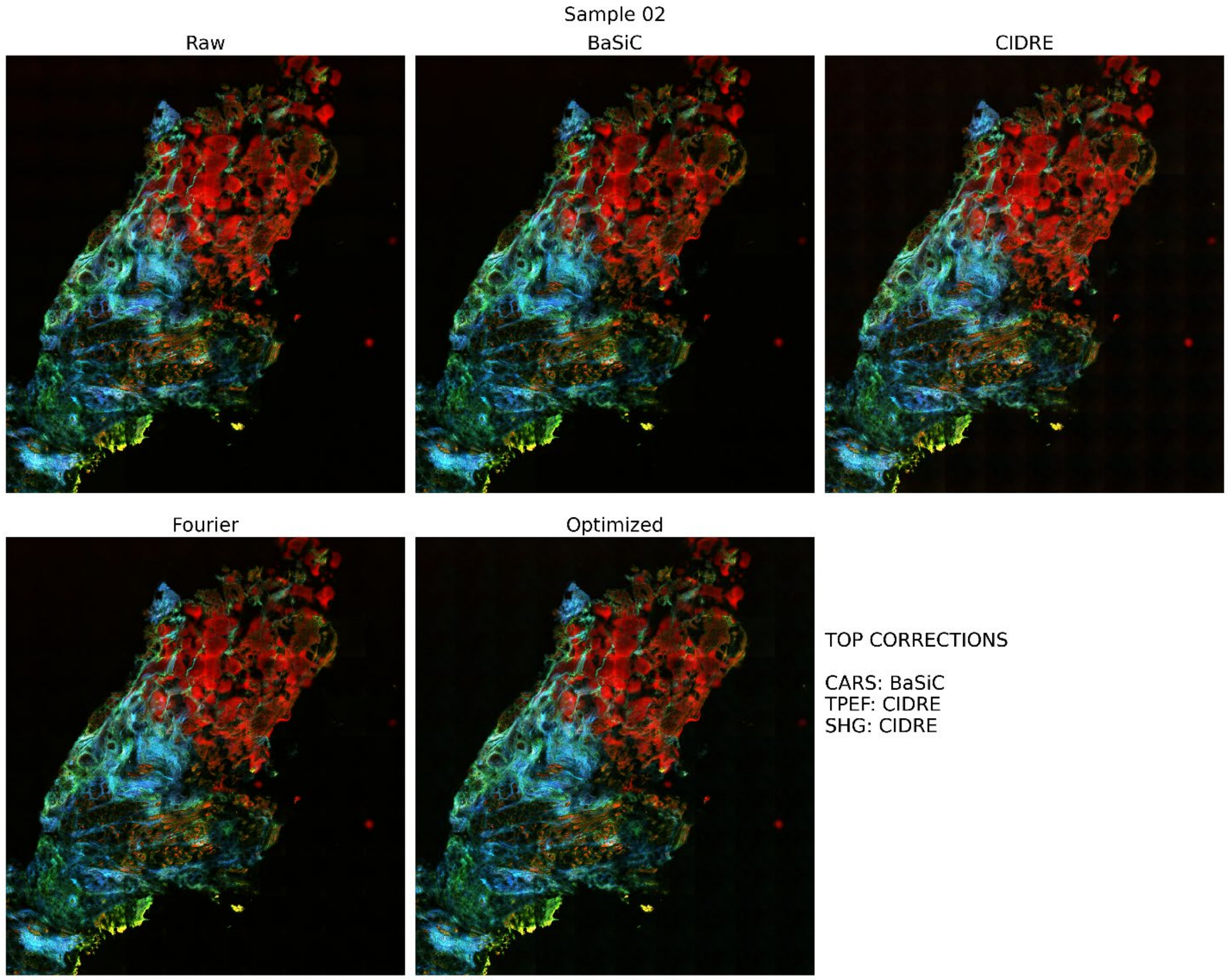

Supplementary Fig. 7 – Single-method corrections and EVEN output (optimized) for Sample 02. *Red channel: coherent anti-Stokes Raman scattering (CARS); green channel: two-photon excited fluorescence (TPEF); blue channel: second harmonic generation (SHG).*

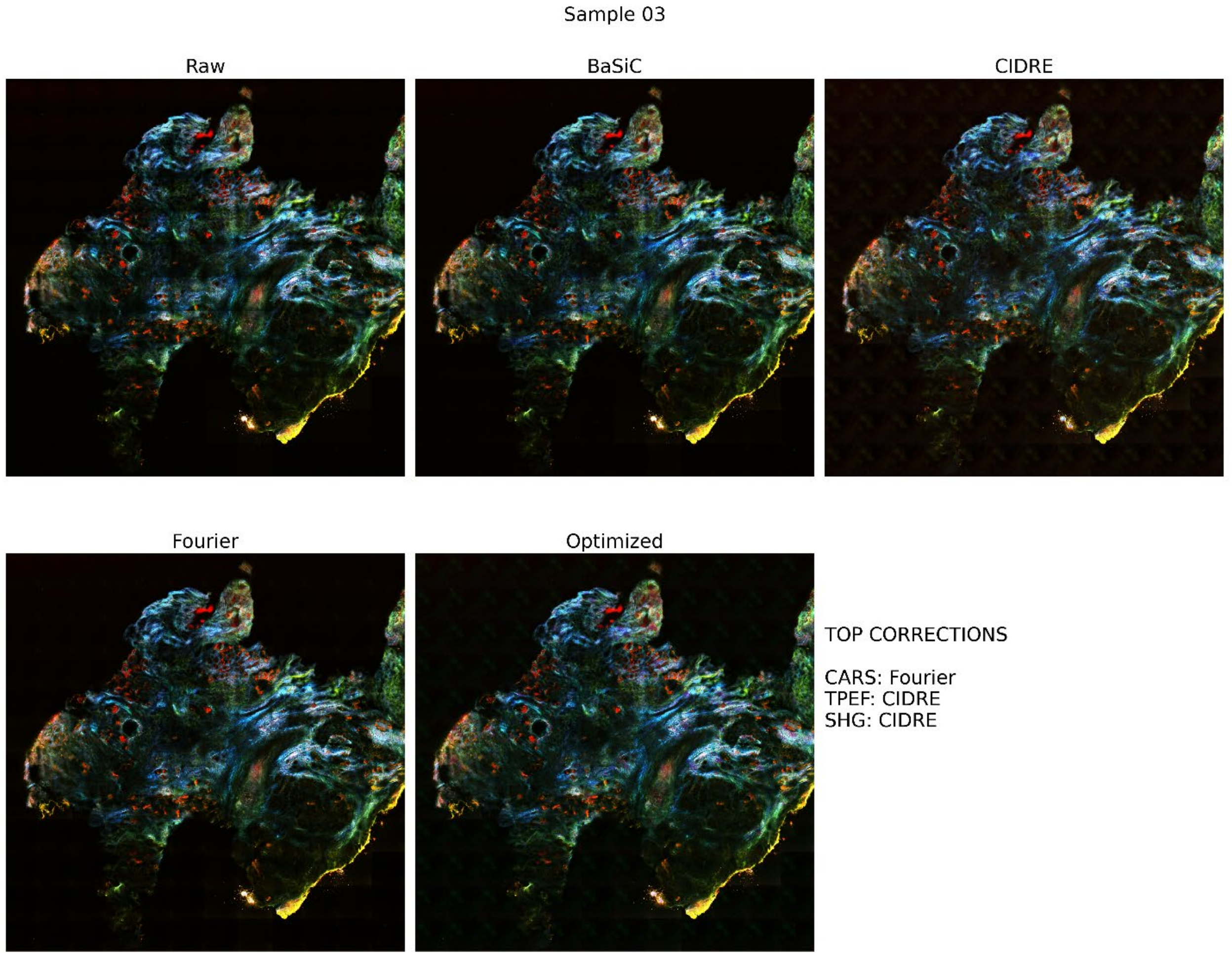

Supplementary Fig. 8 – Single-method corrections and EVEN output (optimized) for Sample 03. *Red channel: coherent anti-Stokes Raman scattering (CARS); green channel: two-photon excited fluorescence (TPEF); blue channel: second harmonic generation (SHG).*

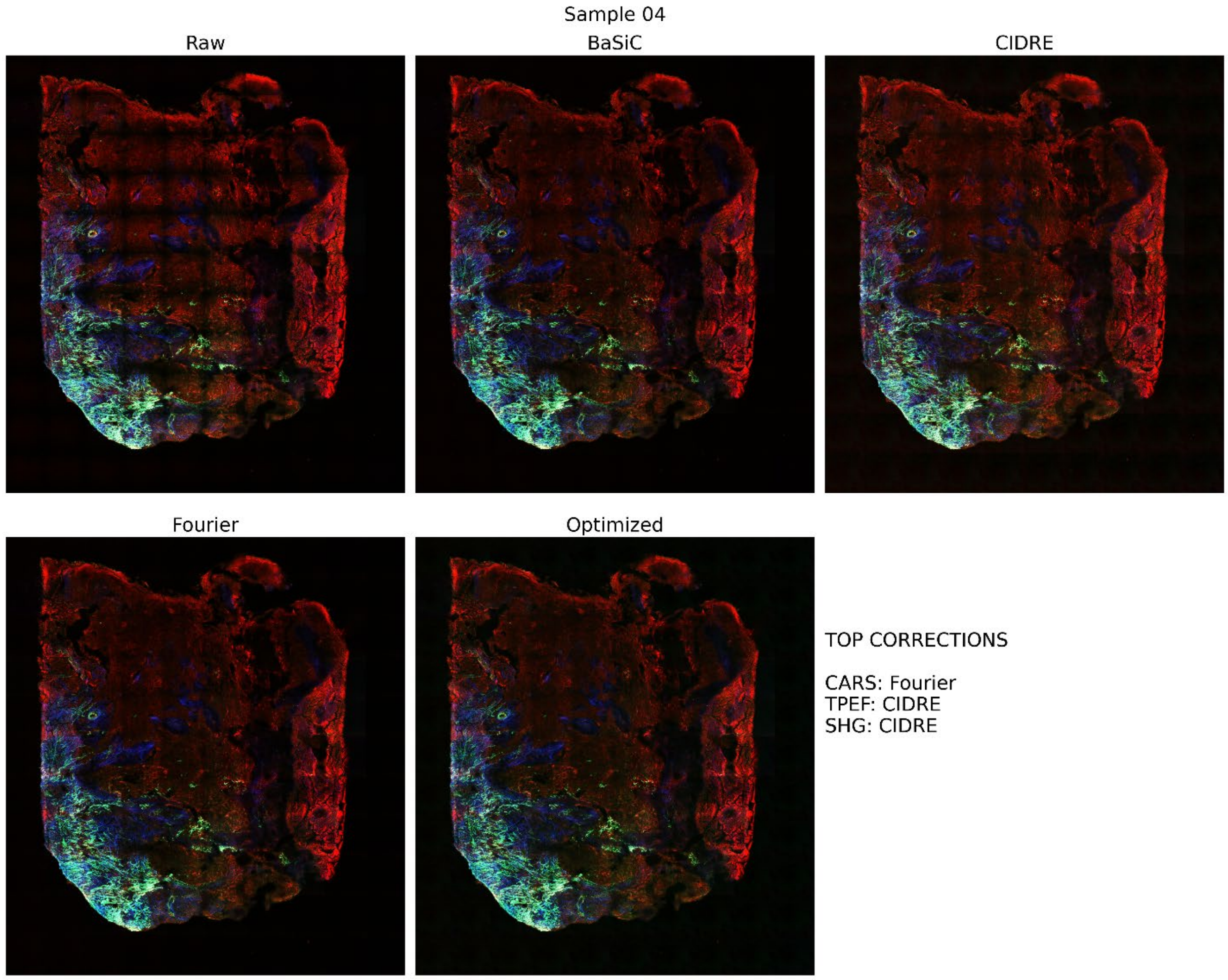

Supplementary Fig. 9 – Single-method corrections and EVEN output (optimized) for Sample 04. *Red channel: coherent anti-Stokes Raman scattering (CARS); green channel: two-photon excited fluorescence (TPEF); blue channel: second harmonic generation (SHG).*

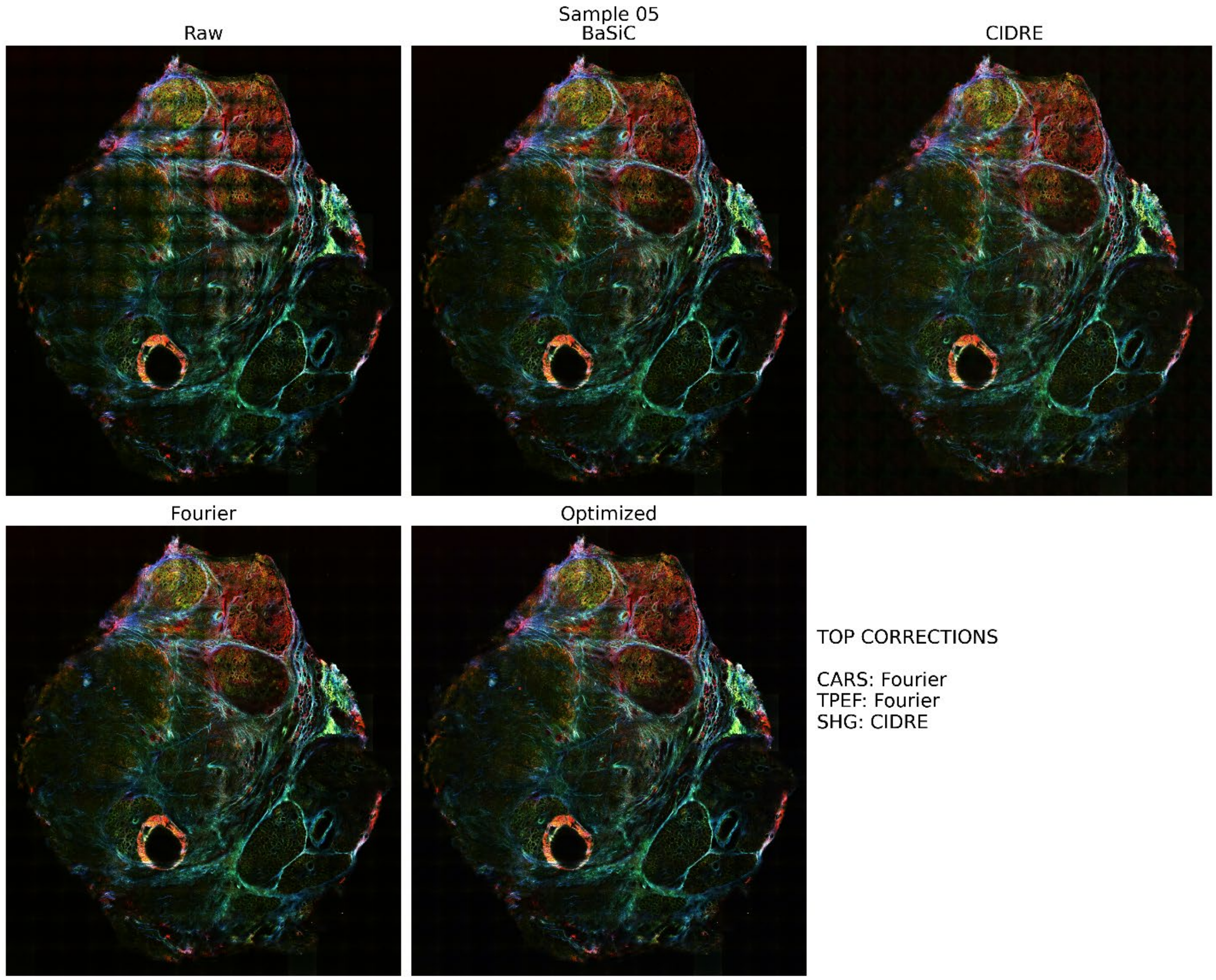

Supplementary Fig. 10 – Single-method corrections and EVEN output (optimized) for Sample 05. *Red channel: coherent anti-Stokes Raman scattering (CARS); green channel: two-photon excited fluorescence (TPEF); blue channel: second harmonic generation (SHG).*

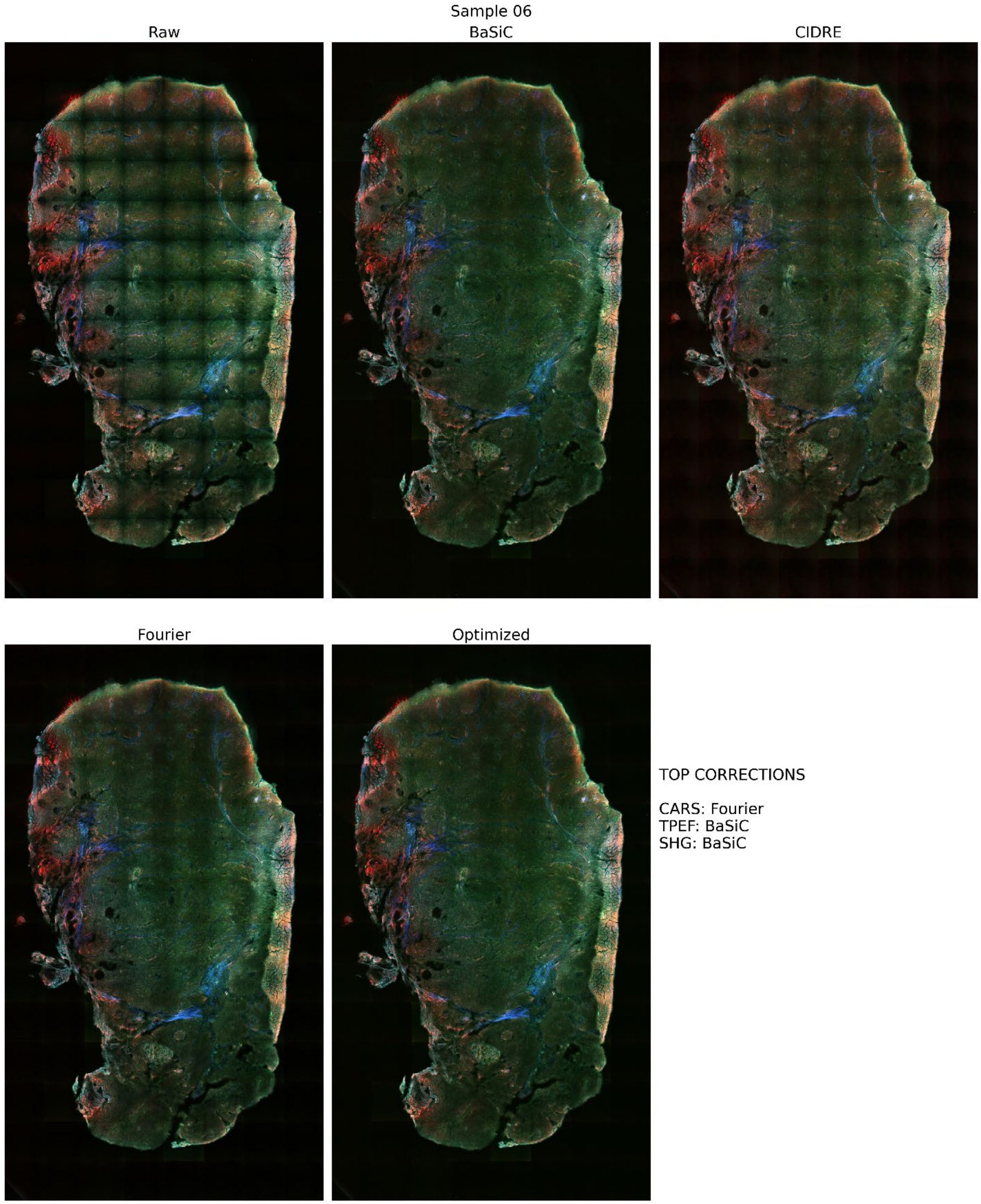

Supplementary Fig. 11 – Single-method corrections and EVEN output (optimized) for Sample 06. *Red channel: coherent anti-Stokes Raman scattering (CARS); green channel: two-photon excited fluorescence (TPEF); blue channel: second harmonic generation (SHG).*

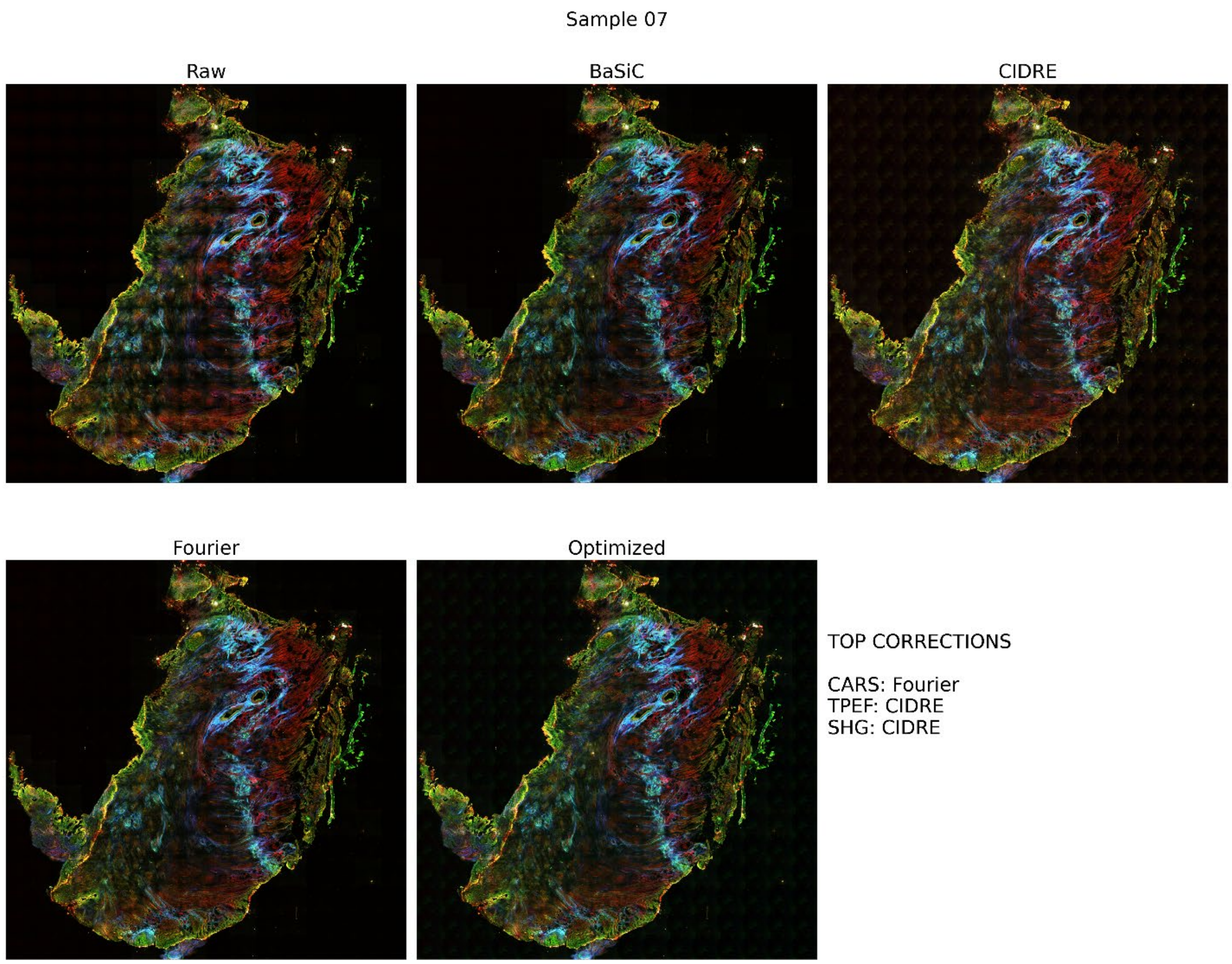

Supplementary Fig. 12 – Single-method corrections and EVEN output (optimized) for Sample 07. *Red channel: coherent anti-Stokes Raman scattering (CARS); green channel: two-photon excited fluorescence (TPEF); blue channel: second harmonic generation (SHG).*

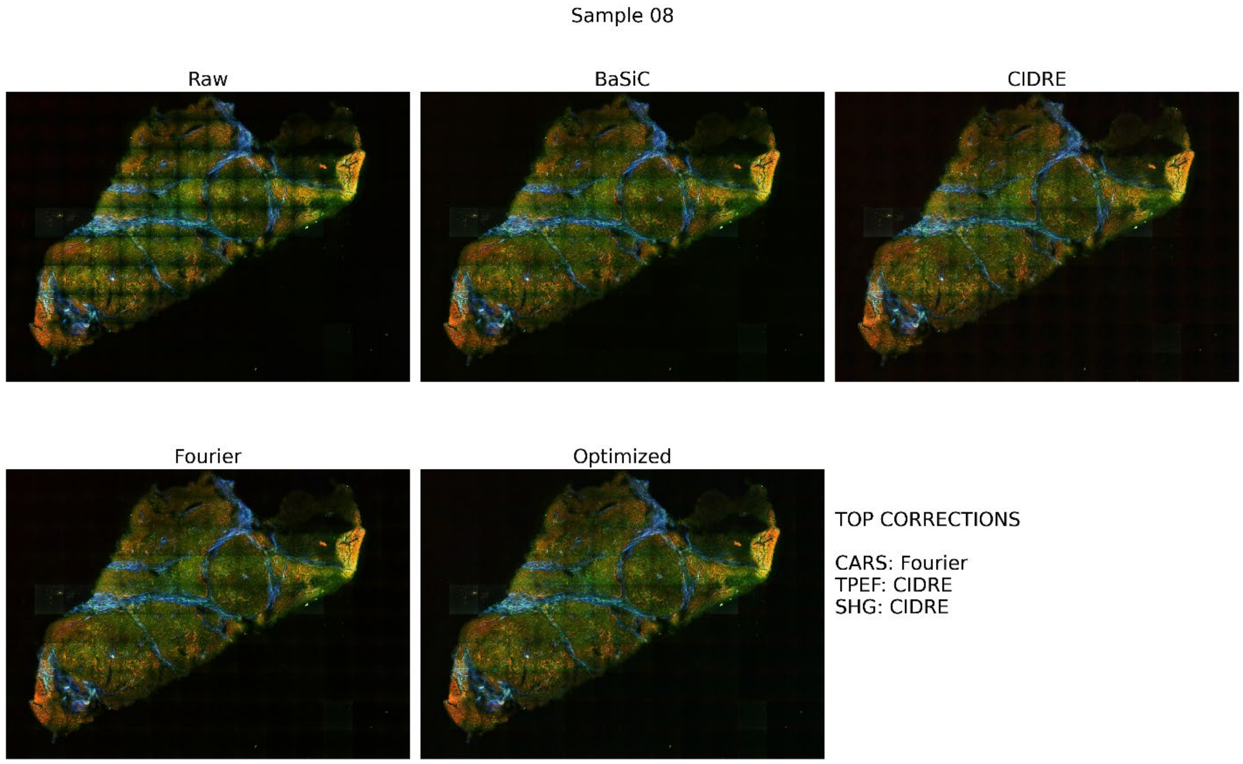

Supplementary Fig. 13 – Single-method corrections and EVEN output (optimized) for Sample 08. *Red channel: coherent anti-Stokes Raman scattering (CARS); green channel: two-photon excited fluorescence (TPEF); blue channel: second harmonic generation (SHG).*

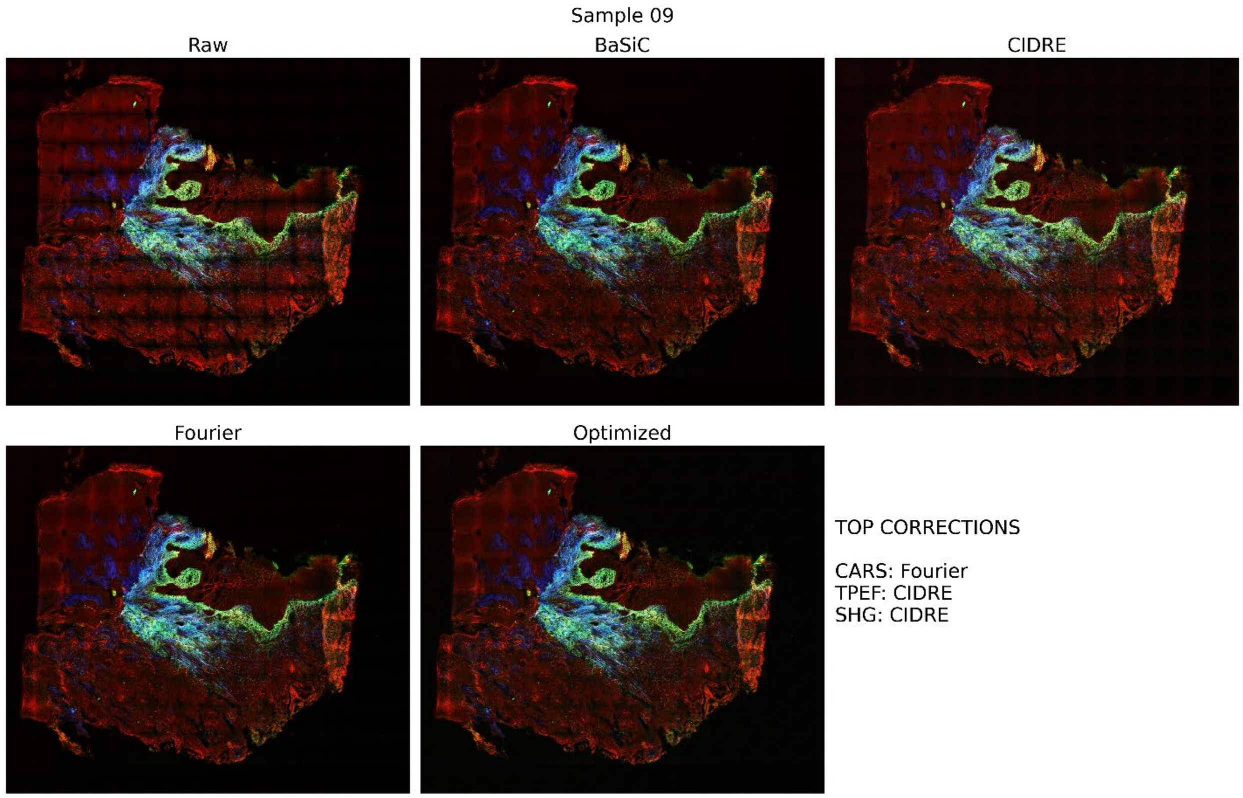

Supplementary Fig. 14 – Single-method corrections and EVEN output (optimized) for Sample 09. *Red channel: coherent anti-Stokes Raman scattering (CARS); green channel: two-photon excited fluorescence (TPEF); blue channel: second harmonic generation (SHG).*

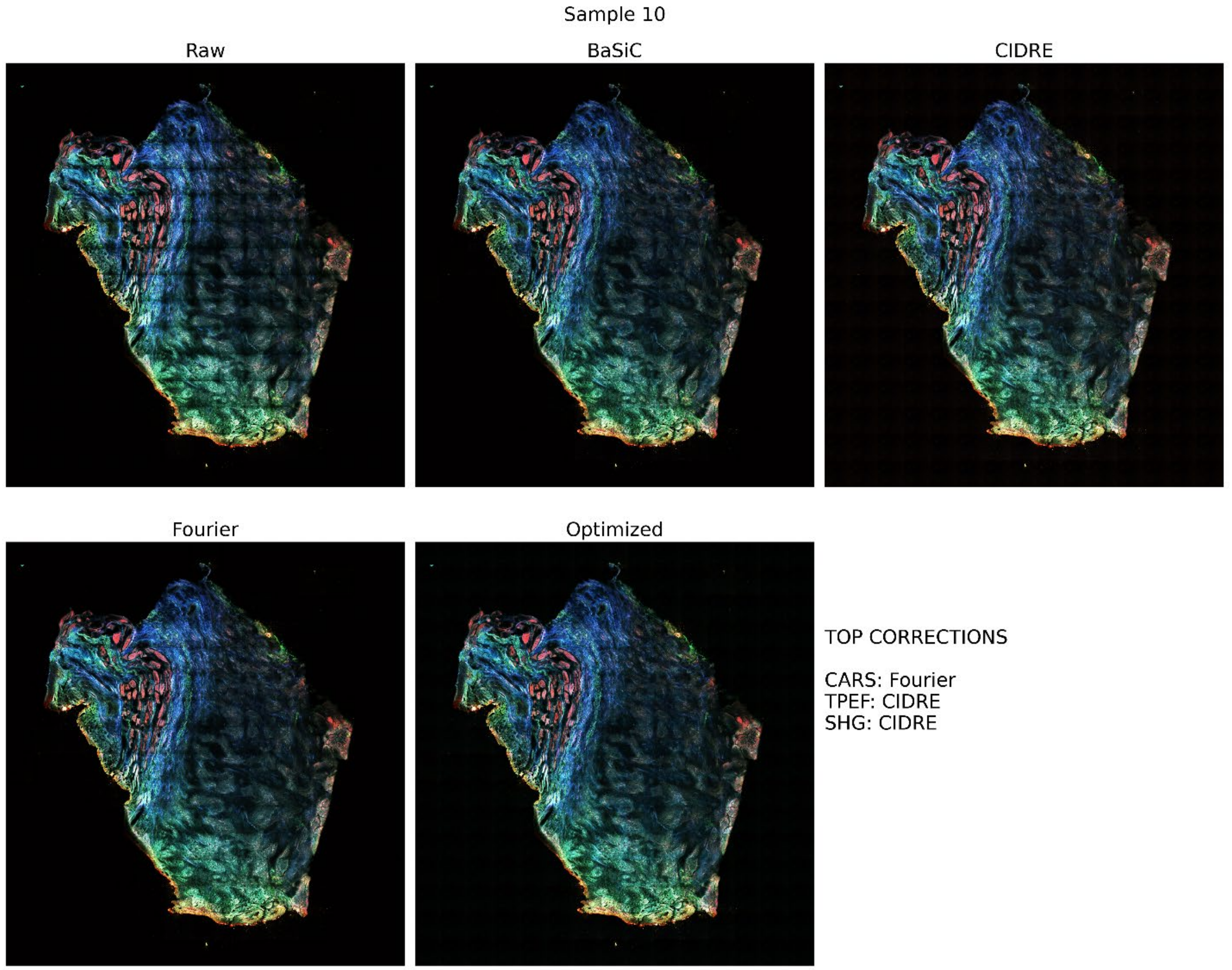

Supplementary Fig. 15 – Single-method corrections and EVEN output (optimized) for Sample 10. *Red channel: coherent anti-Stokes Raman scattering (CARS); green channel: two-photon excited fluorescence (TPEF); blue channel: second harmonic generation (SHG).*

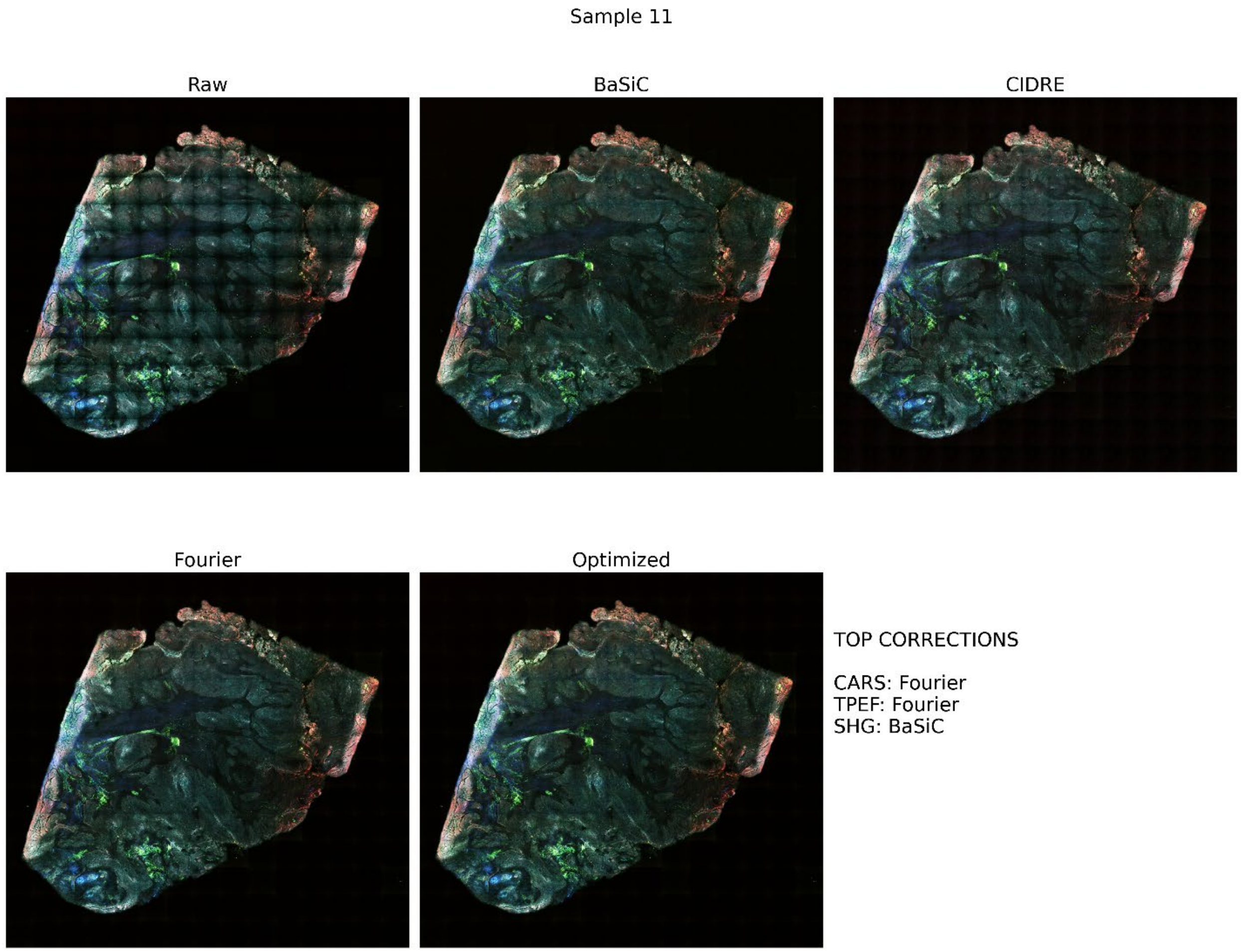

Supplementary Fig. 16 – Single-method corrections and EVEN output (optimized) for Sample 11. *Red channel: coherent anti-Stokes Raman scattering (CARS); green channel: two-photon excited fluorescence (TPEF); blue channel: second harmonic generation (SHG).*

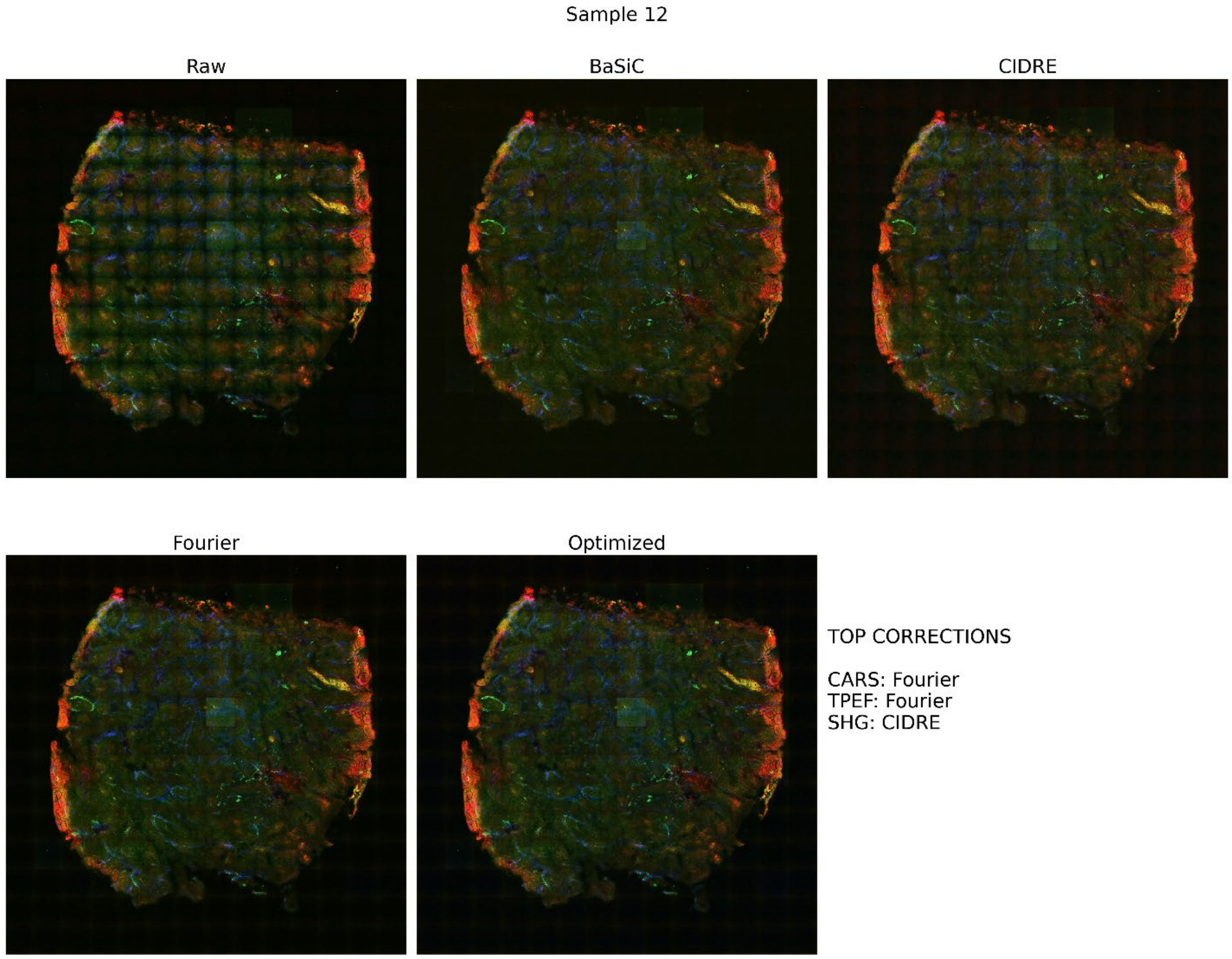

Supplementary Fig. 17 – Single-method corrections and EVEN output (optimized) for Sample 12. *Red channel: coherent anti-Stokes Raman scattering (CARS); green channel: two-photon excited fluorescence (TPEF); blue channel: second harmonic generation (SHG).*

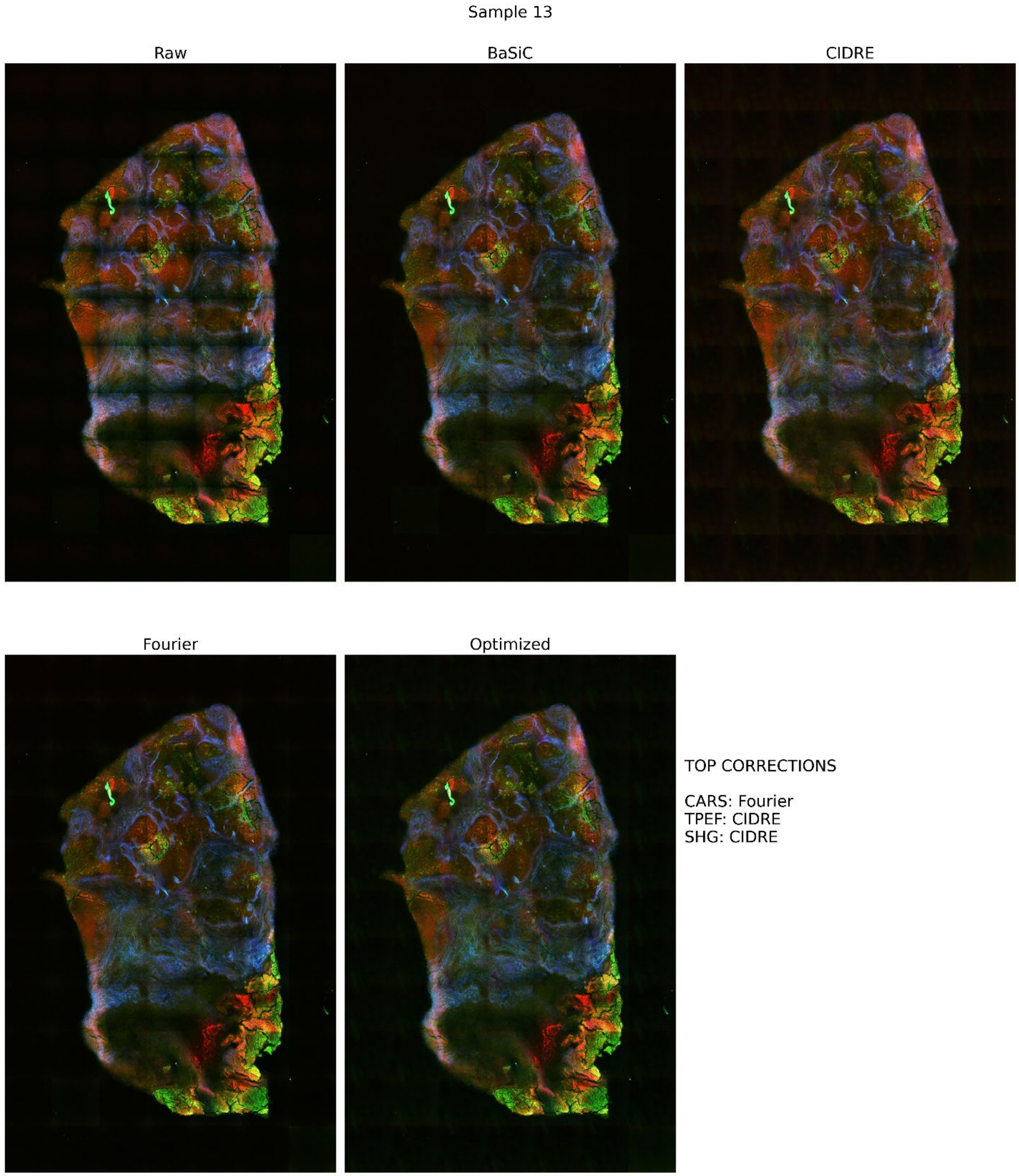

Supplementary Fig. 18 – Single-method corrections and EVEN output (optimized) for Sample 13. *Red channel: coherent anti-Stokes Raman scattering (CARS); green channel: two-photon excited fluorescence (TPEF); blue channel: second harmonic generation (SHG).*

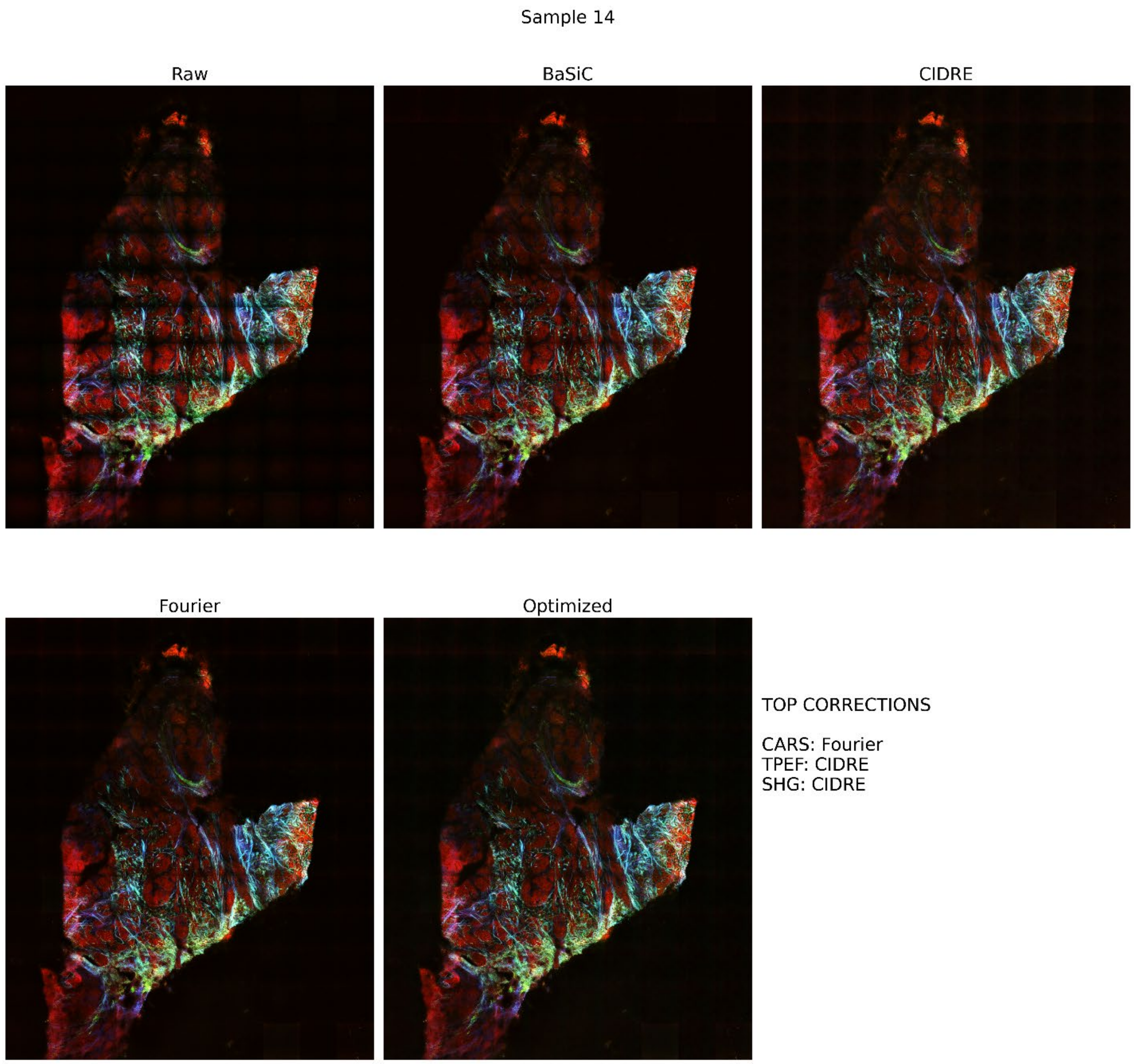

Supplementary Fig. 19 – Single-method corrections and EVEN output (optimized) for Sample 14. *Red channel: coherent anti-Stokes Raman scattering (CARS); green channel: two-photon excited fluorescence (TPEF); blue channel: second harmonic generation (SHG).*

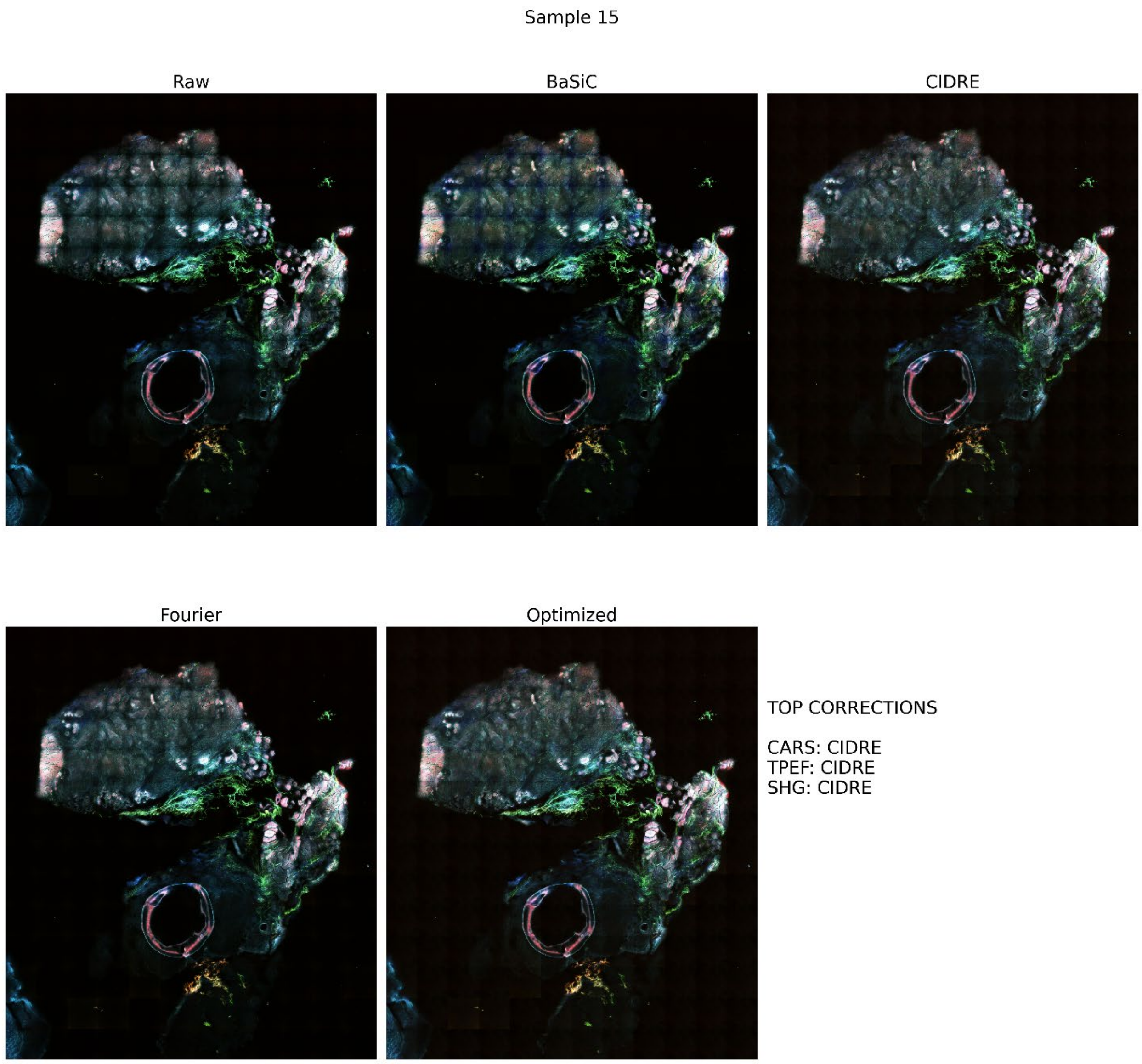

Supplementary Fig. 20 – Single-method corrections and EVEN output (optimized) for Sample 15. *Red channel: coherent anti-Stokes Raman scattering (CARS); green channel: two-photon excited fluorescence (TPEF); blue channel: second harmonic generation (SHG).*

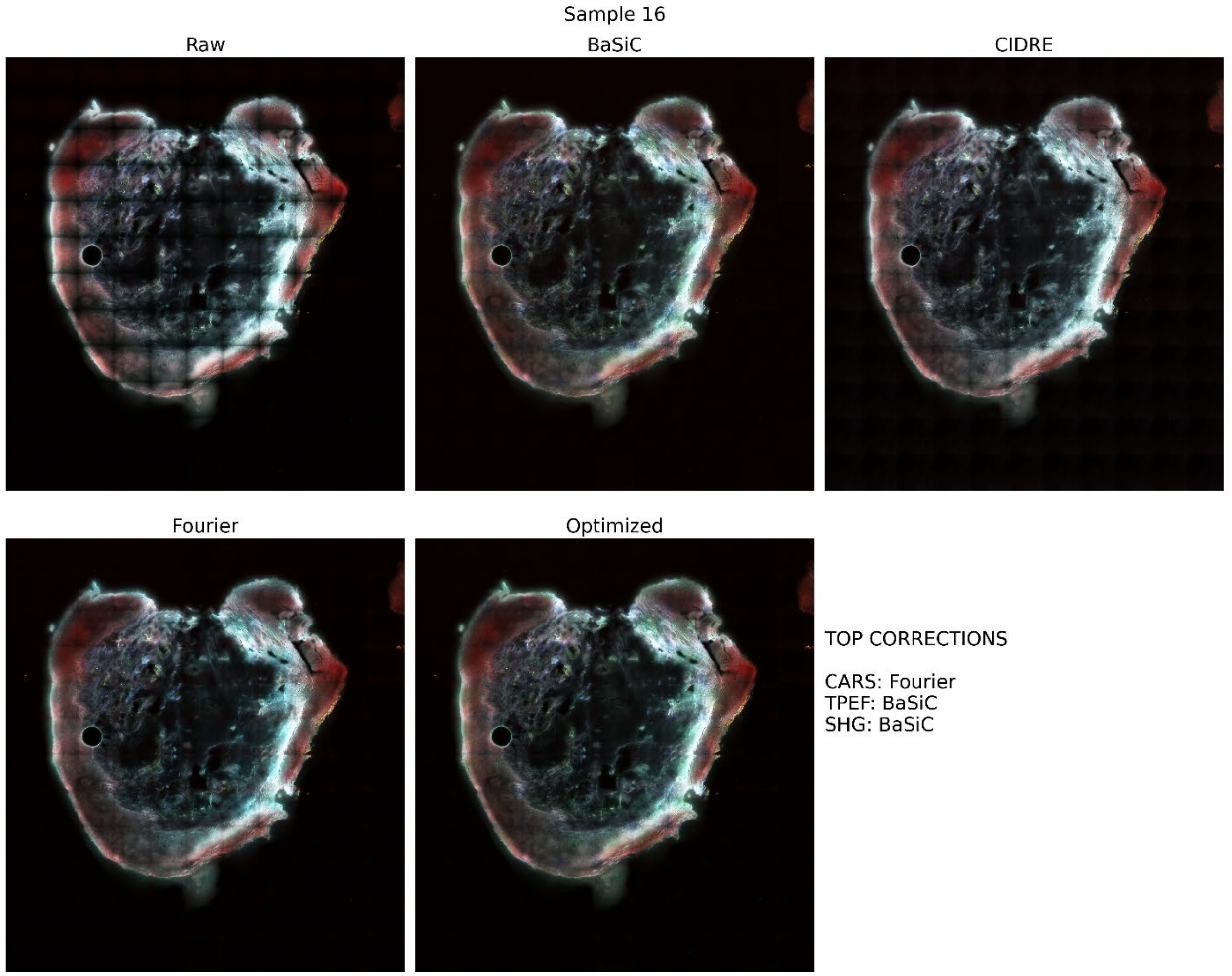

Supplementary Fig. 21 – Single-method corrections and EVEN output (optimized) for Sample 16. *Red channel: coherent anti-Stokes Raman scattering (CARS); green channel: two-photon excited fluorescence (TPEF); blue channel: second harmonic generation (SHG).*

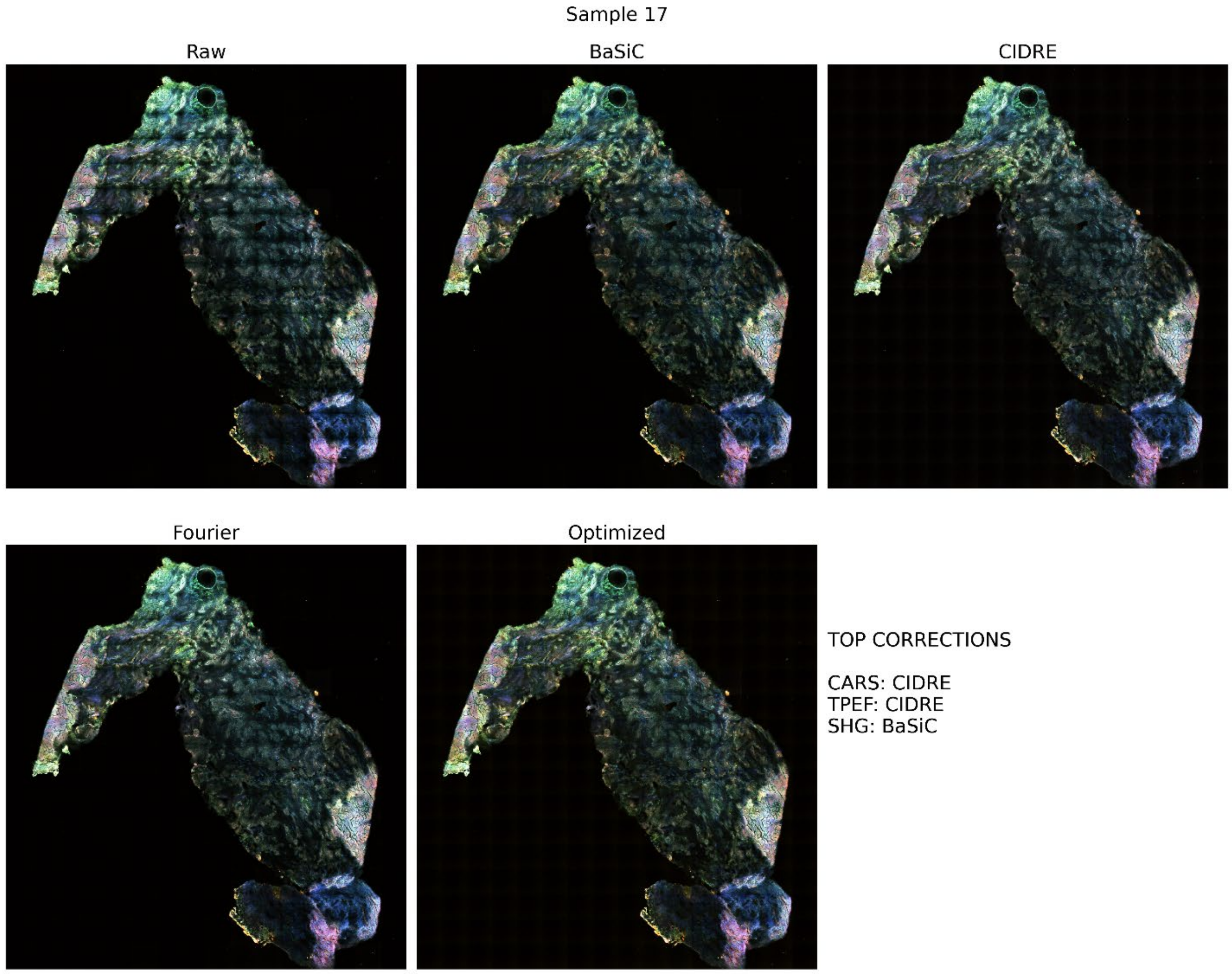

Supplementary Fig. 22 – Single-method corrections and EVEN output (optimized) for Sample 17. *Red channel: coherent anti-Stokes Raman scattering (CARS); green channel: two-photon excited fluorescence (TPEF); blue channel: second harmonic generation (SHG).*

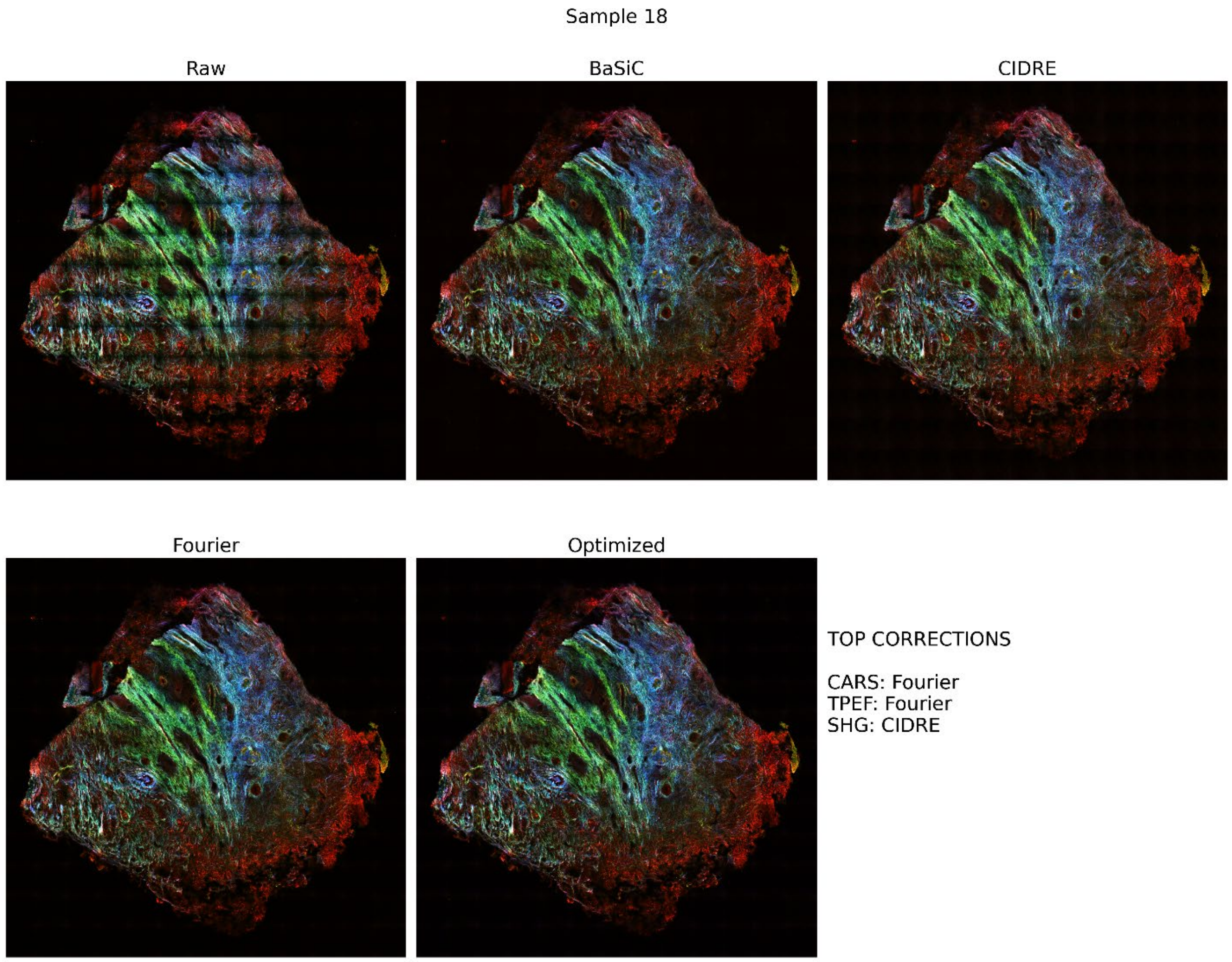

Supplementary Fig. 23 – Single-method corrections and EVEN output (optimized) for Sample 18. *Red channel: coherent anti-Stokes Raman scattering (CARS); green channel: two-photon excited fluorescence (TPEF); blue channel: second harmonic generation (SHG).*

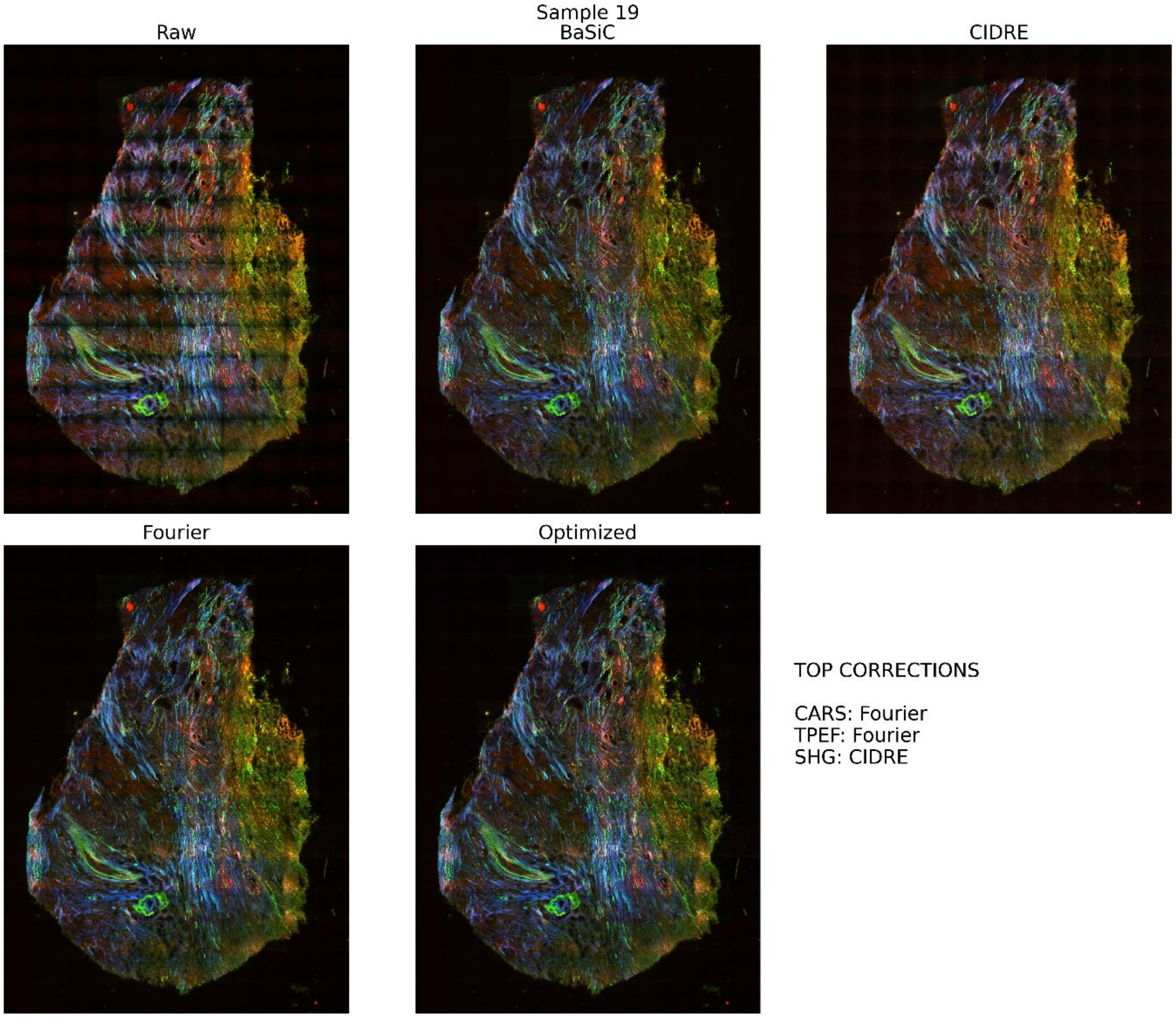

Supplementary Fig. 24 - Single-method corrections and EVEN output (optimized) for Sample 19. *Red channel: coherent anti-Stokes Raman scattering (CARS); green channel: two-photon excited fluorescence (TPEF); blue channel: second harmonic generation (SHG).*

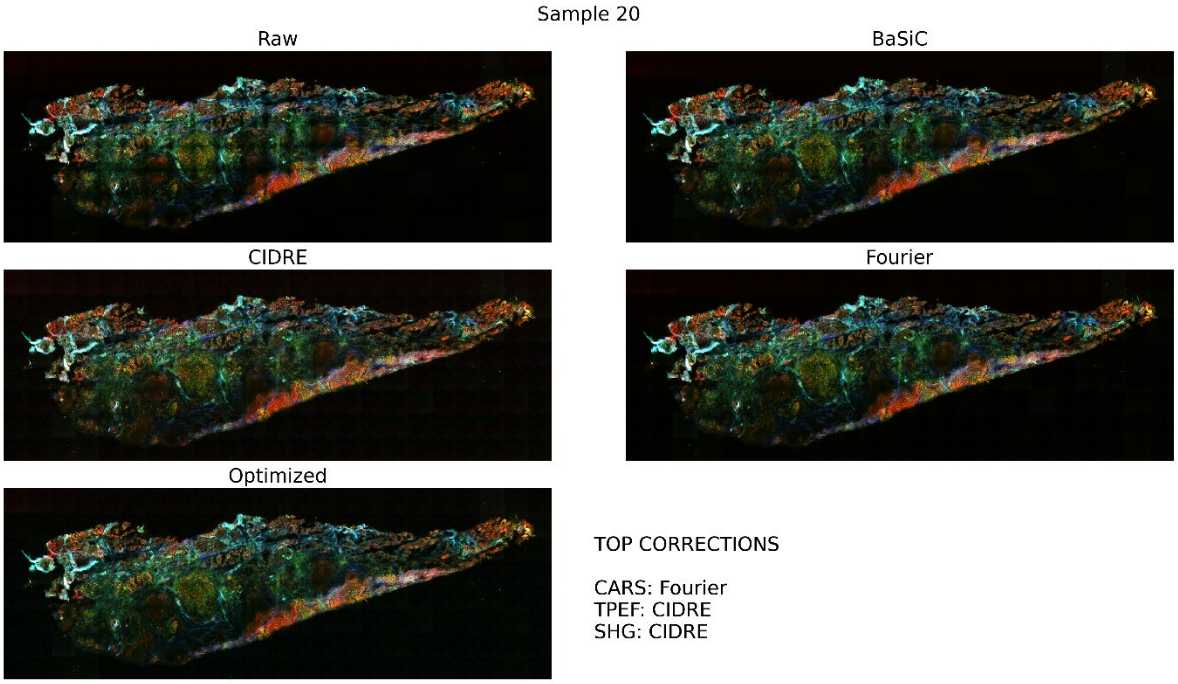

Supplementary Fig. 25 – Single-method corrections and EVEN output (optimized) for Sample 20. *Red channel: coherent anti-Stokes Raman scattering (CARS); green channel: two-photon excited fluorescence (TPEF); blue channel: second harmonic generation (SHG).*

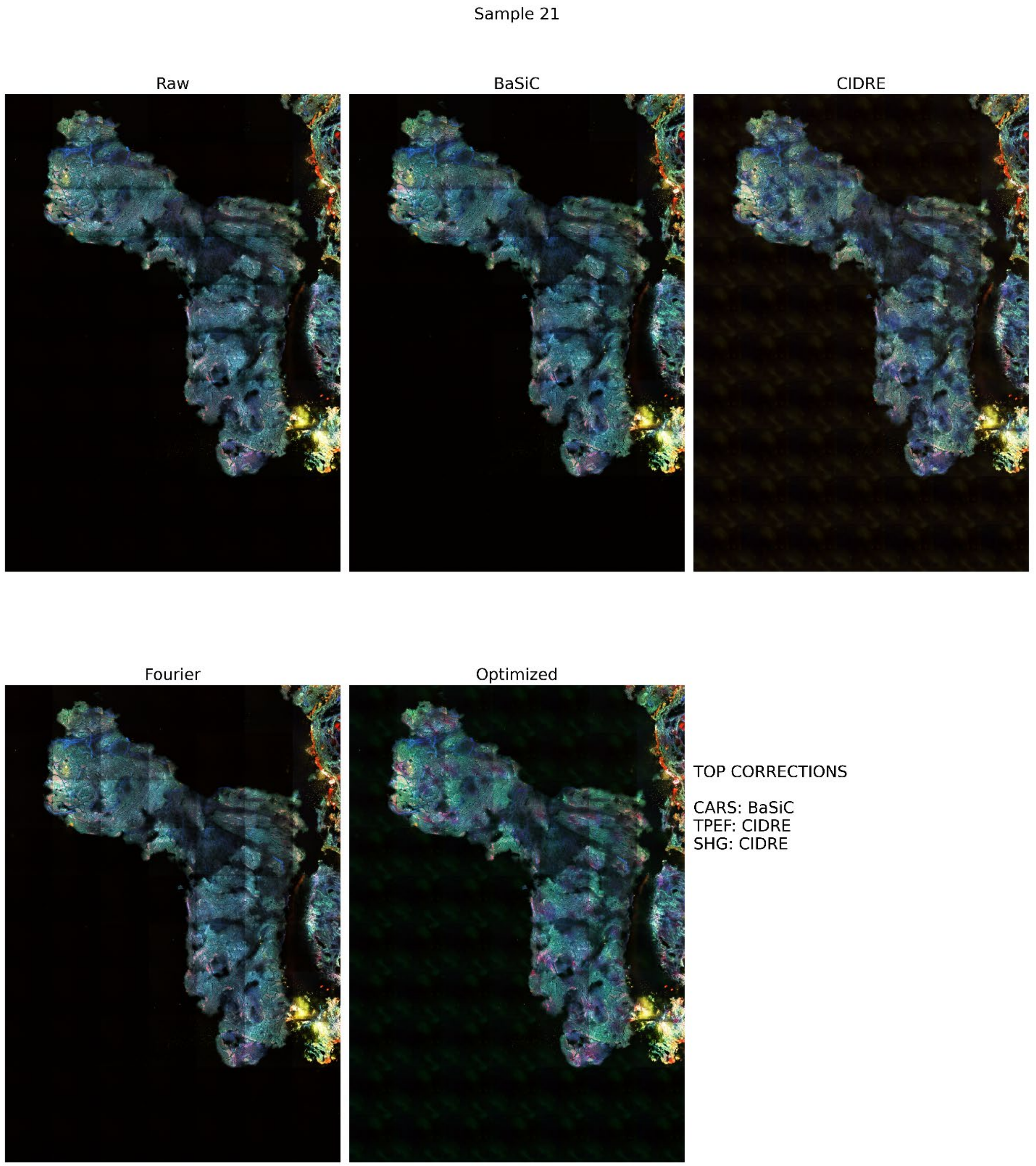

Supplementary Fig. 26 – Single-method corrections and EVEN output (optimized) for Sample 21. *Red channel: coherent anti-Stokes Raman scattering (CARS); green channel: two-photon excited fluorescence (TPEF); blue channel: second harmonic generation (SHG).*

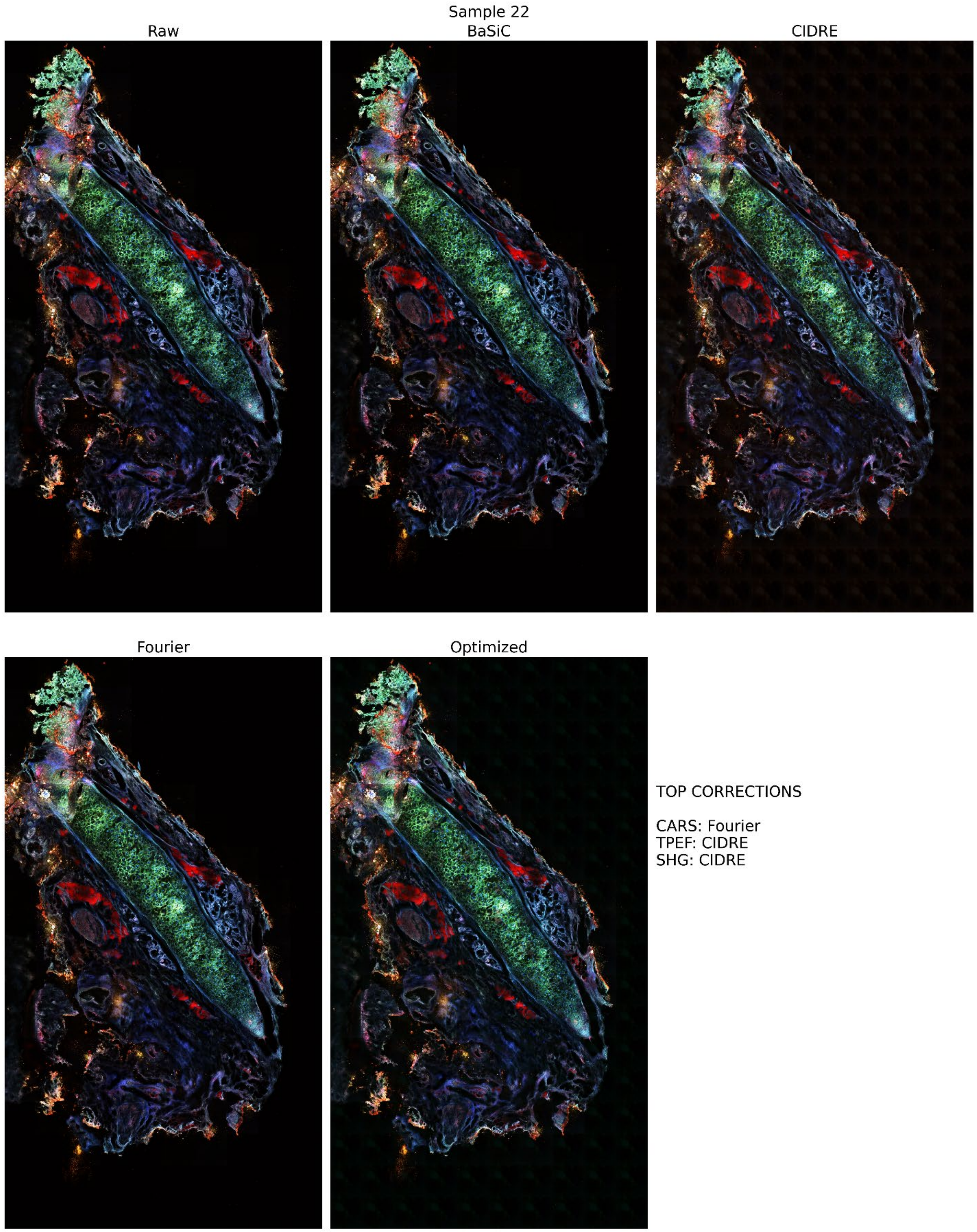

Supplementary Fig. 27 – Single-method corrections and EVEN output (optimized) for Sample 22. *Red channel: coherent anti-Stokes Raman scattering (CARS); green channel: two-photon excited fluorescence (TPEF); blue channel: second harmonic generation (SHG).*

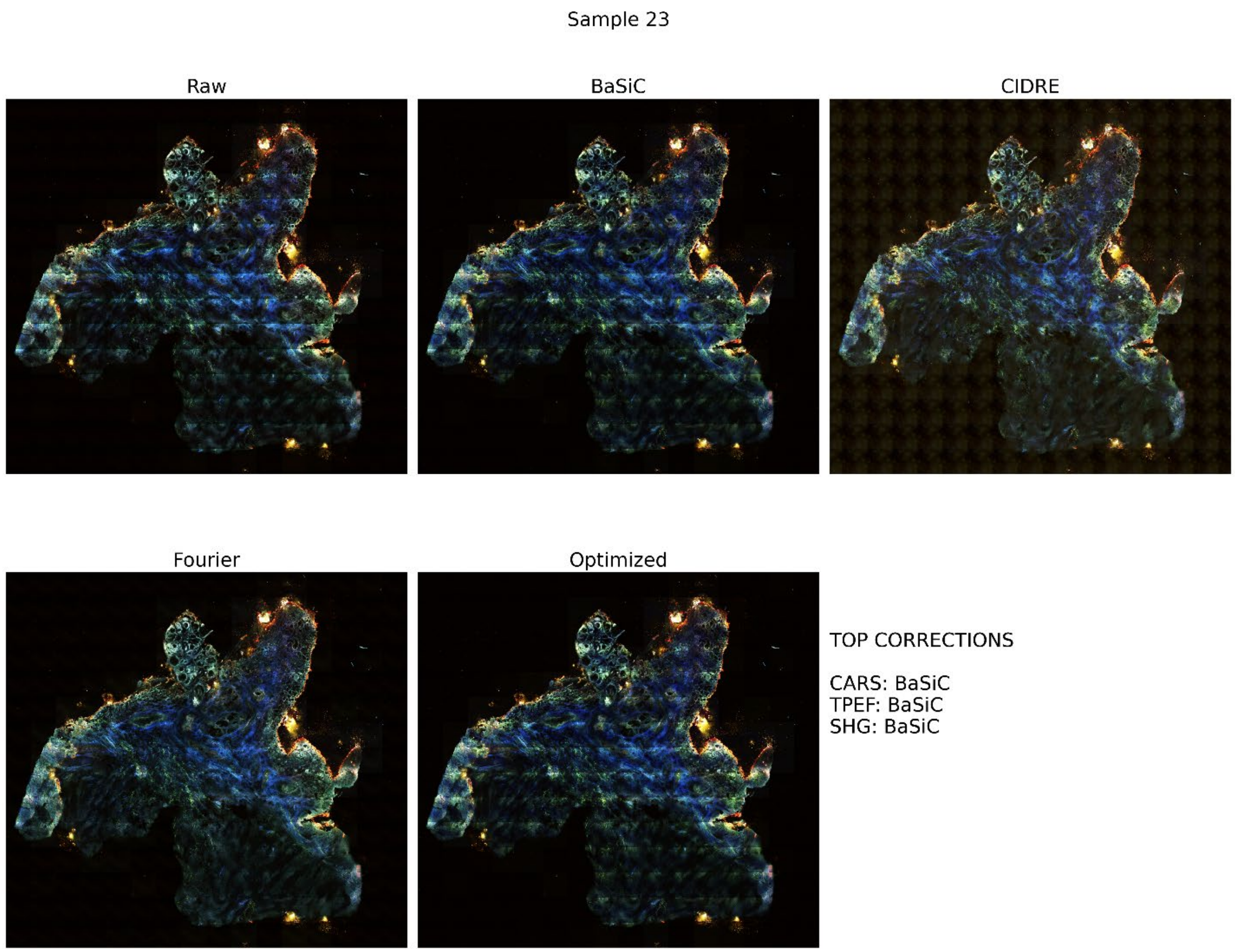

Supplementary Fig. 28 – Single-method corrections and EVEN output (optimized) for Sample 23. *Red channel: coherent anti-Stokes Raman scattering (CARS); green channel: two-photon excited fluorescence (TPEF); blue channel: second harmonic generation (SHG).*

Supplementary Fig. 29: Optimization of multimodal measurements of stained cells

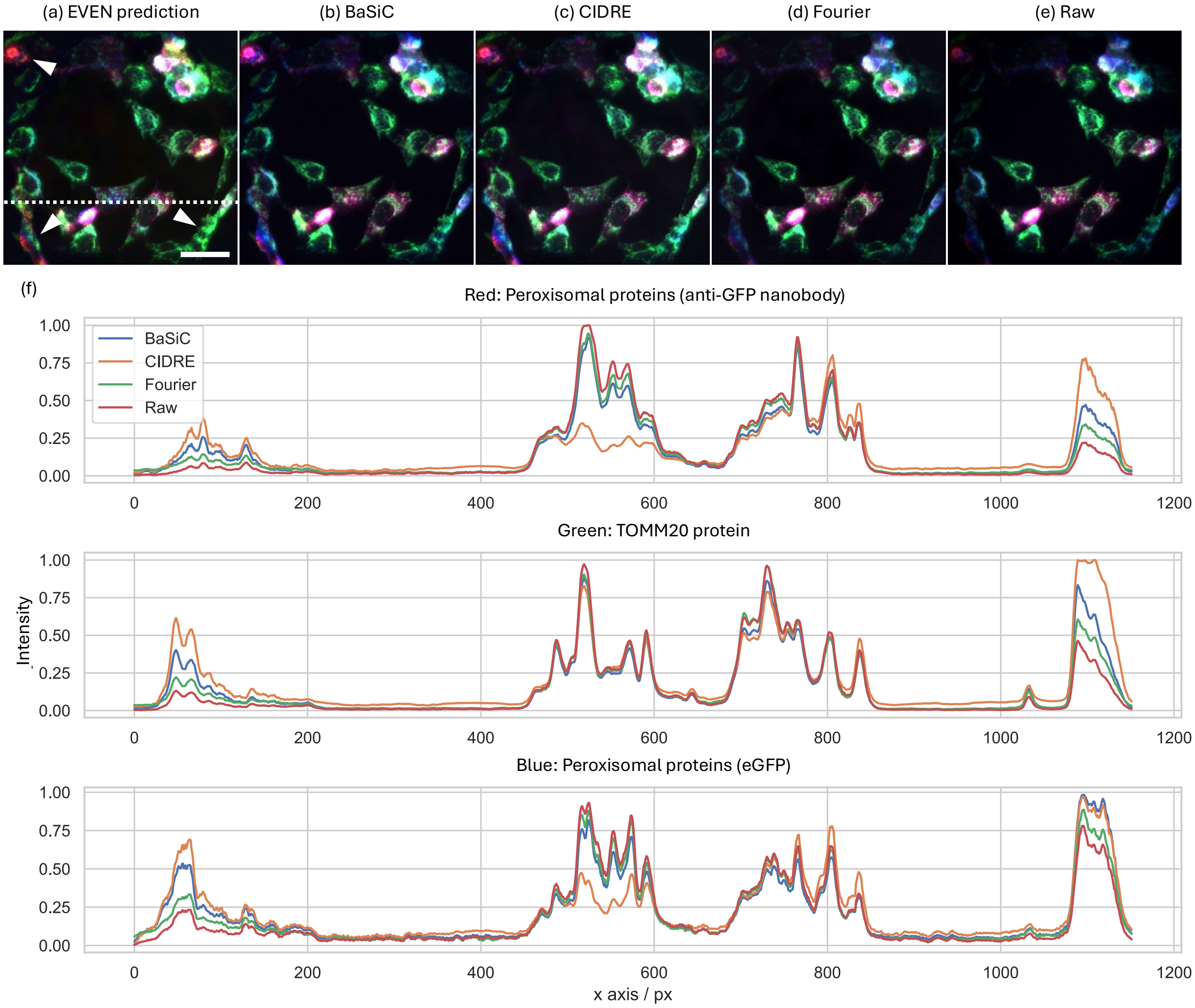

Supplementary Fig. 29 – ***Evaluation and Enhancement of experimental measurements of stained HEK293 cells (Prediction dataset 2).*** *(a-e) Single tile of the composite image, from Fig. 5 of the main manuscript. From left to right, panels show the optimized image predicted by EVEN, the corrections obtained by BaSiC[3], CIDRE[4], and Fourier[1], and the raw image. The white arrows in panel (a) show relevant features enhanced by the correction methods. Scale bar: 50 µm. (f) Intensity profiles along the dashed white line in panel (a) for all correction methods. Each plot shows the profiles along a single channel, as indicated by the titles. Profiles are traced for images rescaled between 0 and 1. EVEN enables the selection of single channels characterized by the best reduction of periodic artifacts and a more uniform intensity distribution in single tiles, and generates a reliable output image in terms of individual contribution of single channels. EVEN selects for the red and green channels the CIDRE correction, that shows the highest enhancement of features at the edges of the profile (see also the red spot highlighted in the top-left edge of the corrected tile). For the blue channel, EVEN selects the Fourier method, which provides good signal enhancement at the edges of single tiles, as shown in panel (f), but also good removal of the periodic grid in the composite image and an equalization of the global profile (Fig. 5, panel (b)).*

Supplementary Fig. 30: Comparison of different experimental configurations for the same sample

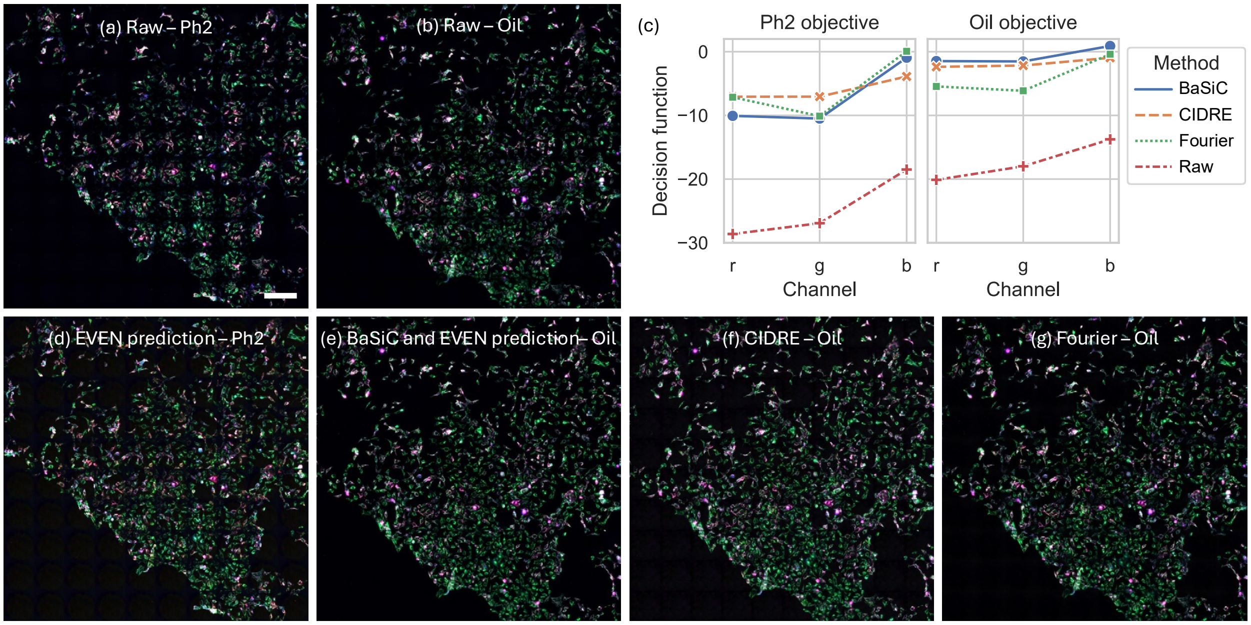

Supplementary Fig. 30 – ***Evaluation and Enhancement of stained HEK293 cells measured with oil-immersion objective****. The same sample of Fig. 5 is measured with an Oil-immersion objective to reduce uneven illumination during the measurement process. (a) Raw image measured with Ph2 objective. Red: peroxisomal proteins (anti-GFP nanobody); Green: TOMM20 protein; Blue: peroxisomal proteins (eGFP). (b) Improved image measured by oil objective, that shows a reduced mosaicking artifact. (c) Comparison of the decision scores predicted by EVEN for the raw and corrected versions of single channels of the Ph2 (left) and Oil (right) measurements. (d) EVEN prediction for Ph2-objective measurement. (e) EVEN prediction for the Oil-objective measurement, that coincides with BaSiC[3] correction for all channels. (f-g) CIDRE[4] and Fourier[1] corrections for the Oil-objective measurement. EVEN is utilized here in two stages of the experimental pipeline: first, EVEN detects the higher quality of the raw image acquired with the oil-immersion objective; then, it is utilized to generate an optimized corrected image starting from the best raw measurement.*

Supplementary Fig. 31: EVEN score and Cellpose cell count for measurements of stained cells

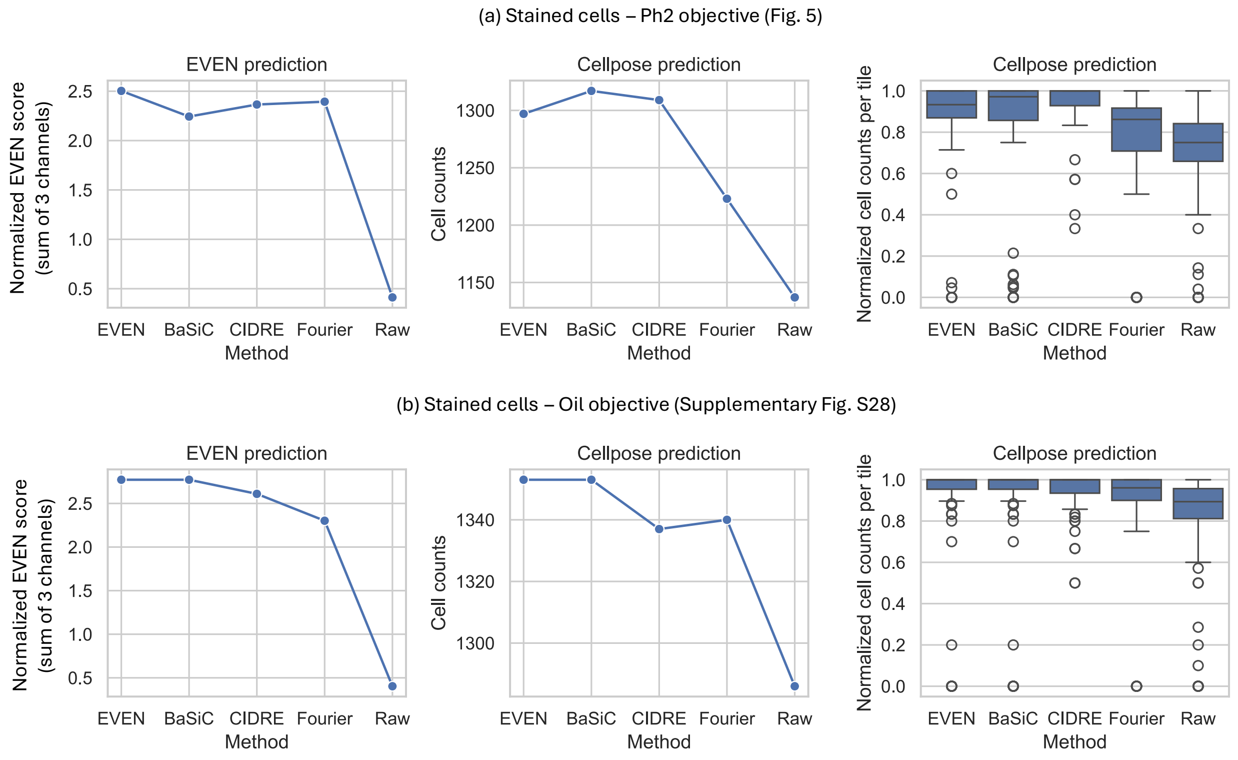

Supplementary Fig. 31 - ***EVEN score and Cellpose[5,6] segmentation for multi-channel measurements of stained cells acquired with Ph2 objective (a) and Oil objective (b).*** *Left column: normalized EVEN score computed for the EVEN optimization, single-method corrections and raw images. The reported score is the sum of the maximum normalized score of the three channels. Central column: number of cells counted by Cellpose for full-size multi-channel corrections. Right column: normalized cells count predicted separately for each tile by Cellpose; the boxplots show the statistics computed on n = 100 tiles composing each image. Removal of uneven illumination enables the identification of a higher number of cells. Boxplots indicate median (middle line), first and third quartile (box); whiskers extend until the 1.5 interquartile range and outliers are marked by single points.*

Supplementary Fig. 32: Optimization of a multimodal measurement of a cell culture

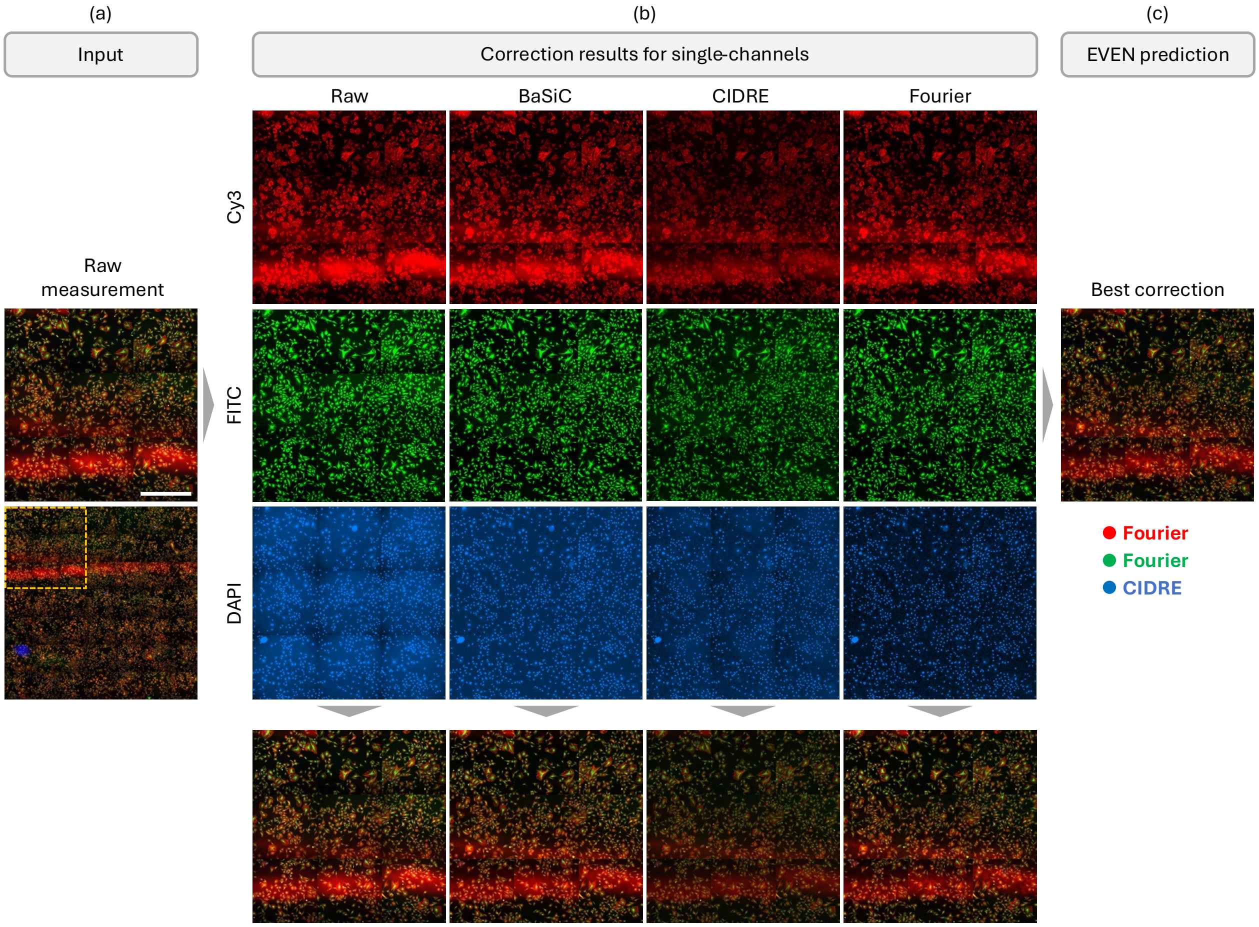

*Supplementary Fig. 32 – **Evaluation and Enhancement of experimental measurements of cell culture** (prediction dataset 3, available from a previous work[3]). (a) Raw input measurement with strong uneven illumination. The figure shows the top-left crop extracted from the yellow frame in the whole image. (b) The three channels are corrected independently by BaSiC, CIDRE, and Fourier method. For a better visualization of the differences between the corrections, the contrast of the single channels is tuned (only for this panel) by setting the maximum value to half of the dynamic range. Correction methods show a different performance for different channels, that is evident also in the multimodal corrections obtained by single methods (bottom row). (c) The optimized correction is predicted by EVEN. For this specific measurement, the Fourier method is selected as the best correction for the Cy3 and FITC channels, while CIDRE is selected for DAPI channel. The optimized image shows stronger reduction of uneven illumination and mitigation of possible colour distortion issues (such as the strong intensity reduction in the Cy3 channel caused by CIDRE) compared to the single-method cases. Scale bar: 1 mm.*

Supplementary Fig. 33: Automatic quality ranking of a single channel measurement of a mouse brain slice

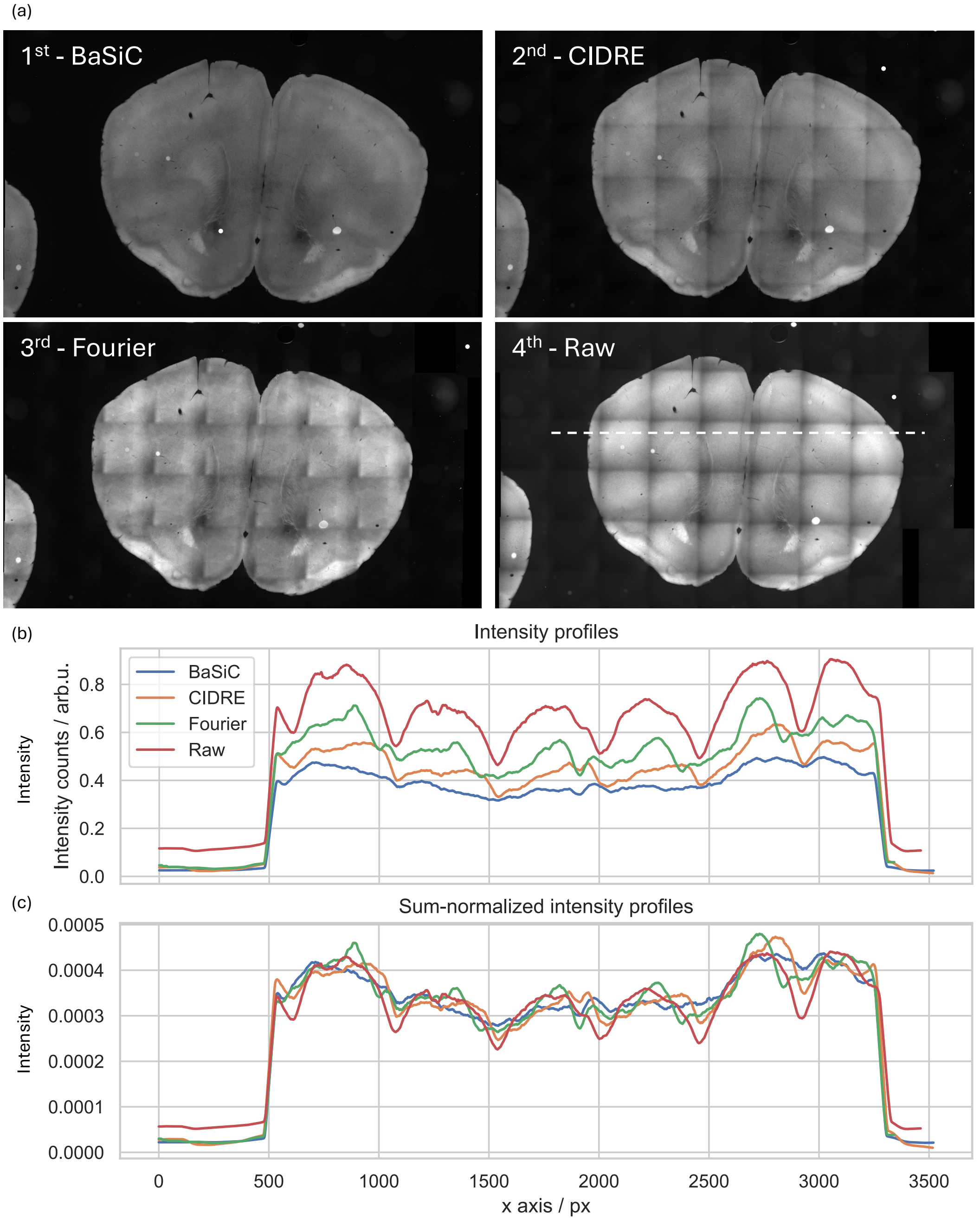

*Supplementary Fig. 33 – **Evaluation and Enhancement of experimental measurements of a mouse brain slice** (prediction dataset 4, available from a previous work[3]). (a) EVEN is utilized to predict a quality ranking for the single-channel measurement. The prediction is applied before computation of the custom stitching provided by the authors. (b) Intensity profiles traced along the white dashed line in the raw image of panel (a), smoothed with an average filter of size 50. The corrected images show a reduction of the background intensity and of the periodic artifact: Fourier method shows a residual periodic peak introduced by the correction and visible in panel (a). CIDRE reduces uneven illumination with a weak residual artifact, whereas BaSiC shows the best performance. (c) The sum-normalized rescaling of the intensity profiles demonstrates that the BaSiC output distributes the profile energy uniformly, without relevant losses of information.*

Supplementary Fig. 34: Automatic quality ranking of a timelapse movie of differentiating cells

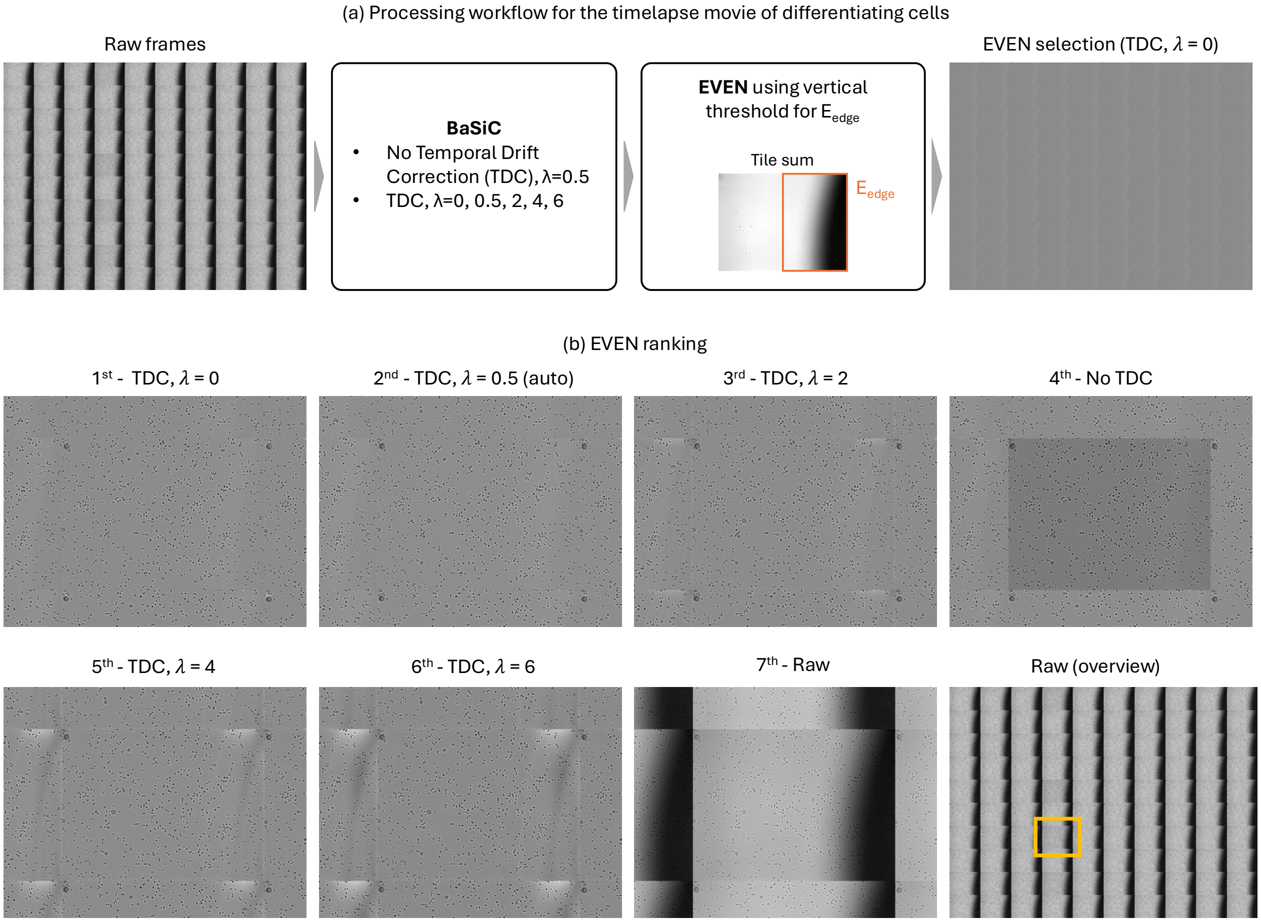

Supplementary Fig. 34 – ***EVEN optimization of BaSiC hyperparameters for a timelapse movie with temporal drift of the intensity.*** *(a) The differentiation of mouse hematopoietic stem cells is measured in a timelapse movie with 100 frames (prediction dataset 5, available from a previous work[3]). The frames show shading on the right side and a temporal change of the intensity. Therefore, they require an optimized correction with BaSiC, exploiting the Temporal Drift Correction (TDC) option and tuning the λ parameter. We applied BaSiC without TDC (λ=0.5), and with TDC (λ=0, 0.5, 2, 4, 6), then we generated a composite image by stitching the frames and we applied EVEN evaluation. Due to lateral shading, we selected a vertical threshold to identify the shaded area in the edge energy ratio. (b) Quality ranking computed by EVEN. For a clear visualization of the residual artifacts, we show the cropped region highlighted by a yellow frame in the bottom-right panel. EVEN places at the top of the ranking corrections with no or minimal residual artifact, then the correction without TDC, where uneven illumination is removed successfully, and finally the corrections with strong residual shading.*

Supplementary Fig. 35: Optimization of a small multimodal composite image

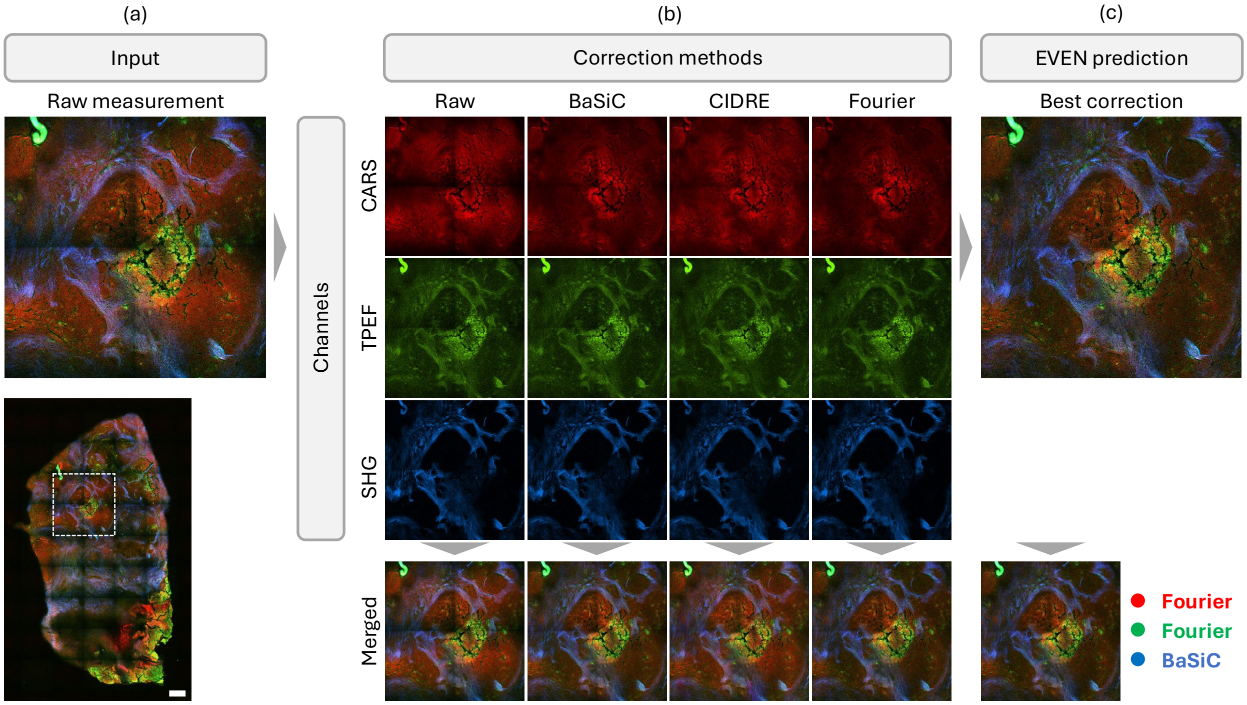

*Supplementary Fig. 35 - **Evaluation and Enhancement of a multimodal image of human head and neck tissue slice on a small 2x2 tile region.** One of the multimodal images of prediction dataset 1 is automatically optimized by EVEN. (a) Raw input measurement with strong uneven illumination. The small image region is extracted from the region marked by the white frame in the full measurement. (b) The three channels are corrected independently with three different correction methods: BaSiC[3], CIDRE[4], Fourier[1]. Due to different intensity distribution in the channels, that are generated by the different morphological features of the different tissue components, the correction methods show a different performance for different channels. Indeed, multimodal corrections obtained by single methods (bottom row) are different and show residual artifacts. (c) The optimized correction is predicted by EVEN after the computation of the quality metrics for each correction of the channels. The evaluation differs from the result of Fig. 4 due to the different properties of this local region of interest. Scale bar: 200 µm.*

Supplementary Table 1: Computational time of correction methods and EVEN

	Processing time / s						
Type of image	BaSiC	CIDRE	Fourier	EVEN: Evaluate	EVEN: Train	EVEN: Predict + optimize	EVEN: Notebook
Single channel	13.61	12.73	0.79 (1.75)	5.53 (1.97)	0.09	0.004	-
3 channels	40.83	38.19	2.37 (5.25)	15.74	0.09	3.89	41.47

Supplementary Table 1 – ***Processing time of correction methods and EVEN for the multimodal image of head and neck tissue of Fig. 4.*** *The column contain, from left to right, the processing time required for different steps of the workflow: BaSiC[3] (Fiji plugin), CIDRE[4] (Fiji plugin), Fourier[1] (function implemented in the public repository, with the internal time to compute the function and, in brackets, the time get the image from the GPU and apply final normalization), Evaluate (computation of the quality metrics), Train (training of the LDA model), Predict + optimize (prediction of the image score and generation of the optimized multimodal image), Notebook (full execution of the workflow notebook of the open source repository). The single-channel row includes the time to process a single-channel image. The EVEN evaluation contains the time to evaluate the raw image and the corrections and, in brackets, the evaluation time for a single image. The prediction time is only 4 ms because no multi-modal optimization must be executed. The 3-channel row contains the correction time for three channels and the EVEN workflow executed on the 12 resulting single-channel images using the notebook in the public repository (https://git.photonicdata.science/elena.corbetta/even/-/blob/main/workflow_even.ipynb).*

Supplementary References

1. Chernavskaia O, Guo S, Meyer T, Vogler N, Akimov D, Heuke S, Heintzmann R, Bocklitz T, Popp J. Correction of mosaicking artifacts in multimodal images caused by uneven illumination. *Journal of Chemometrics* **31**, (2017).
2. Calvarese M, Corbetta E, Contreras J, Bae H, Lai C, Reichwald K, Meyer-Zedler T, Pertzborn D, Muhlig A, Hoffmann F, Messerschmidt B, Guntinas-Lichius O, Schmitt M, Bocklitz T, Popp J. Endomicroscopic AI-driven morphochemical imaging and fs-laser ablation for selective tumor identification and selective tissue removal. *Sci Adv* **10**, eado9721 (2024).
3. Peng T, Thorn K, Schroeder T, Wang L, Theis FJ, Marr C, Navab N. A BaSiC tool for background and shading correction of optical microscopy images. *Nat Commun* **8**, 14836 (2017).
4. Smith K, Li Y, Piccinini F, Csucs G, Balazs C, Bevilacqua A, Horvath P. CIDRE: an illumination-correction method for optical microscopy. *Nat Methods* **12**, 404-406 (2015).
5. Stringer C, Wang T, Michaelos M, Pachitariu M. Cellpose: a generalist algorithm for cellular segmentation. *Nature Methods* **18**, 100--106 (2021).
6. Stringer C, Pachitariu M. Cellpose3: one-click image restoration for improved cellular segmentation. *Nature Methods* **22**, 592-599 (2025).
